# Genetic parameters and parental and early-life effects of boar semen traits

**DOI:** 10.1186/s12711-025-00954-6

**Published:** 2025-02-06

**Authors:** Pedro Sá, Rodrigo M. Godinho, Marta Gòdia, Claudia A. Sevillano, Barbara Harlizius, Ole Madsen, Henk Bovenhuis

**Affiliations:** 1 Animal Breeding and Genomics, Wageningen University and Research, 6700 AH Wageningen, The Netherlands; 2 Topigs Norsvin Research Center B.V., 5216 TZ ‘s-Hertogenbosch, The Netherlands

## Abstract

**Background:**

The objectives of this study were to estimate genetic parameters and studying the influence of early-life and parental factors on the semen traits of boars. The dataset included measurements on 449,966 ejaculates evaluated using a Computer-Assisted Sperm Analysis (CASA) system from 5692 artificial insemination (AI) boars. In total, we considered 16 semen traits measured on fresh semen and 6 sperm motility traits measured on semen after storage. Early-life effects included the dam’s parity, ages of the dam and sire, gestation length, litter size, litter sex ratio, number of piglets born alive, number of litter mates at weaning, rearing length, and weight gain. A repeatability model accounting for effects at collection was used to (1) estimate heritabilities and repeatabilities for semen traits and genetic and phenotypic correlations between traits, (2) test the significance of early-life effects, (3) quantify the contribution of exclusive dam and sire inheritances to the phenotypic variation, i.e., mitochondrial DNA and the Y chromosome, identified using a pedigree-based approach, and (4) quantify the contribution of maternal and paternal environment effects to the phenotypic variation of semen traits.

**Results:**

We reported heritabilities between 0.11 and 0.27 and repeatabilities between 0.20 and 0.65 for semen traits. Semen quality traits showed a skewed distribution, and their transformation significantly reduced their repeatability estimates. Motility traits measured after storage were genetically different from motility traits measured on fresh semen. Early-life had suggestive effects on a limited number of semen traits. Mitochondrial DNA and the Y chromosome did not explain a discerning proportion of the phenotypic variance and the effect of the paternal environment was also negligible. We estimated a significant maternal environment effect predominantly on sperm motility traits, explaining between 2.3 and 4.6% of the phenotypic variance. Including maternal environmental effects in the model reduced heritability estimates for sperm motility traits and total morphological abnormalities.

**Conclusions:**

Our findings indicate that trait transformation has a large effect on repeatability estimates of semen traits. Sperm motility traits measured on fresh semen are genetically different from sperm motility traits measured after storage. Early-life conditions can have an effect on later semen quantity and quality traits. Mitochondrial DNA and Y chromosome inheritances showed no effect on semen traits. Finally, we emphasize the importance of considering maternal effects when analysing semen traits, which results in lower heritability estimates.

**Supplementary Information:**

The online version contains supplementary material available at 10.1186/s12711-025-00954-6.

## Background

Reproduction is a critical determinant of an individual's fitness and is subject to natural selection in wild populations. In humans, a notable concern revolves around the observed recent decline in semen quality, with an increasing trend in morphological abnormalities and decreasing sperm motility [1, 2]. This decline has been attributed to lifestyle factors such as diet, sedentary lifestyles, and environmental pollution [3, 4].

In livestock populations, artificial selection typically includes multiple production and female reproduction traits in breeding goals, with limited emphasis on male reproduction [5]. However, in pig production systems, male reproduction is relevant, especially for transmitting genetic progress from purebred lines to commercial crossbred offspring.

Few studies have identified factors that influence male reproduction in livestock species [6, 7] and limited knowledge exists about the effect of early-life conditions on male reproduction. Likewise, the extent to which maternal and paternal environments or exclusive maternal inheritance, i.e., mitochondrial DNA and exclusive paternal inheritance, i.e. the Y chromosome, have not been studied in the context of the reproductive performance of their male offspring.

In commercial pig breeding, selective breeding strategies demand comprehensive recording of pedigree and other information. Boars undergo continuous monitoring from conception until their introduction into artificial insemination (AI) stations, resulting in meticulously curated information that can be used to characterise the early-life conditions of AI boars.

Once introduced in AI stations, boars undergo routine and continuous semen collection and evaluation of semen quality using automated high throughput phenotyping with Computer-Assisted Sperm Analysis (CASA) systems [8]. CASA systems produce extensive, automated, and highly reproducible measurements for hundreds of semen parameters. The collected data offers excellent opportunities to investigate the impact of early-life conditions of an AI boar on its semen production traits.

The current study aims to estimate genetic parameters for boar semen traits and comprehensively describe maternal influences, including mitochondrial DNA and maternal environmental effects, paternal influences, including Y chromosome and paternal environmental effects, and the role of early-life conditions of the boar on semen production traits measured later in life. We seek to identify factors influencing semen traits in pigs.

## Methods

### Phenotypes

Records on semen traits were generated from fresh and stored ejaculates collected between September 2009 and January 2022 from boars belonging to a commercial synthetic line. Ejaculates were collected as a routine procedure using a semi-automatic collection system and immediately pre-diluted 1:1 using Solusem Bio + (IMV). Evaluation of fresh semen was performed using the IVOS CASA system on pre-diluted ejaculates (Hamilton Thorne Inc., Beverly, MA). After evaluation, ejaculates were fully diluted to an end dose concentration of 1.3–1.5 billion sperm cells and stored in commercial doses of 80 mL at 17 °C. Evaluation of stored semen was performed on random subsets of these commercial doses after one, two, and three days of storage. Finally, microscopy assessments of head abnormalities were recorded on fresh semen. This evaluation was performed by experienced technicians and involved staining a subsample of the ejaculate and identifying and counting sperm cells with abnormal head morphology.

The final dataset consisted of three categories of semen traits, i.e. semen quantity, sperm motility, and sperm morphology. Total number of sperm cells, ejaculate volume and concentration were included as semen quantity traits of an ejaculate. CASA measurements provided data on total and progressive motility of fresh sperm, as well as total morphological abnormalities and individual morphological abnormalities assessments. The latter included total cytoplasmatic droplets, as well as proximal and distal cytoplasmatic droplets, distal midpiece reflex, and bent and coiled tails. Individual morphological abnormalities also included the microscopy data for head and acrosome abnormalities. Based on total normal morphology and total motility assessments, the total number of normal sperm cells and total number of motile sperm cells were also included as semen quantity traits. Phenotypes after storage included measurements of total and progressive motility traits after one, two, and three days of storage at 17 °C.

Records from 44 boars were removed because they had less than three ejaculates. This criterion was particularly relevant for boars that were culled in 2009 and boars that were introduced into semen production in 2022. The final dataset included records from a total of 449,966 ejaculates from 5692 boars, with an average of 79 ejaculates per boar.

### Statistical analyses

In this section, we will start with the introduction of the base model used to estimate genetic parameters. Subsequently, building on this base model, alternative models will be described to address significance of specific effects.

Semen traits were analysed using an animal repeatability model as described in Eq. (1):

1 $$\mathbf{y}=\mathbf{X}{\varvec{\upbeta}}+\mathbf{Z}\mathbf{a}+\mathbf{W}\mathbf{p}+\mathbf{H}\mathbf{h}+ \mathbf{e}$$

where **y** represents a vector with phenotypic observations; **X** is the incidence matrix for the fixed effects at semen collection; **Z**, **W**, and **H** are incidence matrices of random additive genetic, permanent environmental, and herd-year-season of birth effects, respectively; ${\varvec{\upbeta}}$, $\mathbf{a}$, $\mathbf{p}$, and $\mathbf{h}$ are vectors with solutions for fixed (${\varvec{\upbeta}}$) and random effects ($\mathbf{a}$, $\mathbf{p},$ and $\mathbf{h}$). Random additive genetic effects were assumed to be distributed as $\mathbf{a}\sim \text{N}\left(0, \mathbf{A}{\upsigma }_{\text{a}}^{2}\right)$ where **A** is the pedigree-based relationship matrix and ${\upsigma }_{\text{a}}^{2}$ is the additive genetic variance, $\mathbf{p}\sim \text{N}\left(0, \mathbf{I}{\upsigma }_{\text{pe}}^{2}\right)$ where **I** is the identity matrix and ${\upsigma }_{\text{pe}}^{2}$ is the permanent environmental variance, $\mathbf{h}\sim \text{N}\left(0, \mathbf{I}{\upsigma }_{\text{hys}}^{2}\right)$, where **I** is the identity matrix and ${\upsigma }_{\text{hys}}^{2}$ is the variance of herd-year-season of birth, and $\mathbf{e}\sim \text{N}\left(0, \mathbf{I}{\upsigma }_{\text{e}}^{2}\right)$, where **I** is the identity matrix and ${\upsigma }_{\text{e}}^{2}$ is the variance of residual effects.

Analyses were performed using ASREML v.4.2 [9]. The **A** matrix was constructed based on pedigree information that spanned 26 generations and included 17,701 individuals. The complete ancestry up to 8 generations back was known for over 90% of the boars. The distribution of motility traits was right-skewed and a cubic transformation was applied, as previously described for right-skewed production traits in turkeys [10]. The morphology traits were left-skewed and a log-transformation was applied, as previously described for left-skewed semen traits in pigs [11] and for other traits in cattle [12] and horses [13].

Fixed environmental effects at semen collection were included in the base model and in all alternative models. These fixed effects included age of the boar, from 7 to 60 months fitted as a covariate, interval since the last semen collection, fitted as class variable (n = 15 days) between 1 and 15 days, calendar month of collection fitted as class variable (n = 12 months), the person responsible for guiding the boar from the stable to the dummy and collecting the semen fitted as class variable (n = 173 collectors), and the lab technician assessing semen quality with CASA and microscopy fitted as class variable (n = 42 technicians). The effect of age of the boar at collection was fitted in model (1) using a Wilmink function [14], which preliminary analyses showed can describe changes in semen traits with age of the boar in months (t):

2 $$\text{y}=\upbeta 0+\upbeta 1\cdot \text{t}+\upbeta 2\cdot {\text{e}}^{-\text{k}\cdot \text{t}}$$

which includes the trait’s population mean, $\upbeta 0$, a linear term associated with the slope of the curve after reaching an inflection point, $\upbeta 1\cdot \text{t}$, and an exponential term with a coefficient that is associated with the slope of the curve before the inflection point, $\upbeta 2\cdot {\text{e}}^{-\text{k}\cdot \text{t}}$, where k is a coefficient that determines the decay rate of the exponential term. Based on preliminary analyses, k was fixed at 0.45. For all traits, both the linear and exponential terms of age of the boar were significant and included in the model. The exponential term of age was not significant and removed from the model for the transformed and untransformed traits of progressive motility of fresh semen and after one and two days of storage.

### Genetic parameter estimation

Following model (1), variance components, heritabilities (h^2^), and repeatabilities (rep) were estimated for semen traits as:

$${\text{h}}^{2}=\frac{{\upsigma }_{\text{a}}^{2}}{{\upsigma }_{\text{p}}^{2}}$$ and $$\text{rep}=\frac{{\upsigma }_{\text{a}}^{2}+ {\upsigma }_{\text{pe}}^{2}}{{\upsigma }_{\text{p}}^{2}}$$, with $${\upsigma }_{\text{p}}^{2}={\upsigma }_{\text{a}}^{2}+{\upsigma }_{\text{pe}}^{2}+ {\upsigma }_{\text{hys}}^{2}+ {\upsigma }_{\text{e}}^{2}$$

Bivariate analyses using model (1) were used to estimate phenotypic and genetic correlations between semen quantity and transformed motility and morphology traits. The covariance matrices for additive genetic (${\sum }_{\mathbf{a}}$), permanent environment (${\sum }_{\mathbf{p}\mathbf{e}}$), herd-year-season (${\sum }_{\mathbf{h}\mathbf{y}\mathbf{s}}$), and residual (${\sum }_{\mathbf{e}}$) effects for any two traits, T1 and T2, are given as:

$${\sum }_{\mathbf{a}}=\left[\begin{array}{cc}{\upsigma }_{{\text{a}}_{\text{T}1}}^{2}& {\upsigma }_{{\text{a}}_{\text{T}1,\text{T}2}}\\ \text{sym}& {\upsigma }_{{\text{a}}_{\text{T}2}}^{2}\end{array}\right]$$

$${\sum }_{\mathbf{p}\mathbf{e}}=\left[\begin{array}{cc}{\upsigma }_{{\text{pe}}_{\text{T}1}}^{2}& {\upsigma }_{{\text{pe}}_{\text{T}1,\text{T}2}}\\ \text{sym}& {\upsigma }_{{\text{pe}}_{\text{T}2}}^{2}\end{array}\right]$$

$${\sum }_{\mathbf{h}\mathbf{y}\mathbf{s}}=\left[\begin{array}{cc}{\upsigma }_{{\text{hys}}_{\text{T}1}}^{2}& {\upsigma }_{{\text{hys}}_{\text{T}1,\text{T}2}}\\ \text{sym}& {\upsigma }_{{\text{hys}}_{\text{T}2}}^{2}\end{array}\right]$$

$${\sum }_{\mathbf{e}}=\left[\begin{array}{cc}{\upsigma }_{{\text{e}}_{\text{T}1}}^{2}& {\upsigma }_{{\text{e}}_{\text{T}1,\text{T}2}}\\ \text{sym}& {\upsigma }_{{\text{e}}_{\text{T}2}}^{2}\end{array}\right]$$

where ${\upsigma }_{{\text{a}}_{\text{T}1,\text{T}2}}$, ${\upsigma }_{{\text{pe}}_{\text{T}1,\text{T}2}}$, ${\upsigma }_{{\text{hys}}_{\text{T}1,\text{T}2}}$, and ${\upsigma }_{{\text{e}}_{\text{T}1,\text{T}2}}$ describe the additive genetic, permanent environment, herd-year-season, and residual covariances between traits T1 and T2, respectively.

Using the resulting estimates, phenotypic (${\text{r}}_{{\text{p}}_{\text{T}1,\text{T}2}}$), additive genetic (${\text{r}}_{{\text{a}}_{\text{T}1,\text{T}2}}$), permanent environment (${\text{r}}_{{\text{pe}}_{\text{T}1,\text{T}2}}$), herd-year-season of birth (${\text{r}}_{{\text{hys}}_{\text{T}1,\text{T}2}}$), and residual (${\text{r}}_{{\text{e}}_{\text{T}1,\text{T}2}}$) correlations, i.e., (${\text{r}}_{{\text{i}}_{\text{T}1,\text{T}2}}$), between traits T1 and T2 are given by:

$${\text{r}}_{{\text{i}}_{\text{T}1,\text{T}2}}=\frac{{\upsigma }_{{\text{i}}_{\text{T}1,\text{T}2}}}{\sqrt{{\upsigma }_{{\text{i}}_{\text{T}1}}^{2} {\upsigma }_{{\text{i}}_{\text{T}2}}^{2}}}$$

### Early-life conditions

**Fig. 1:**
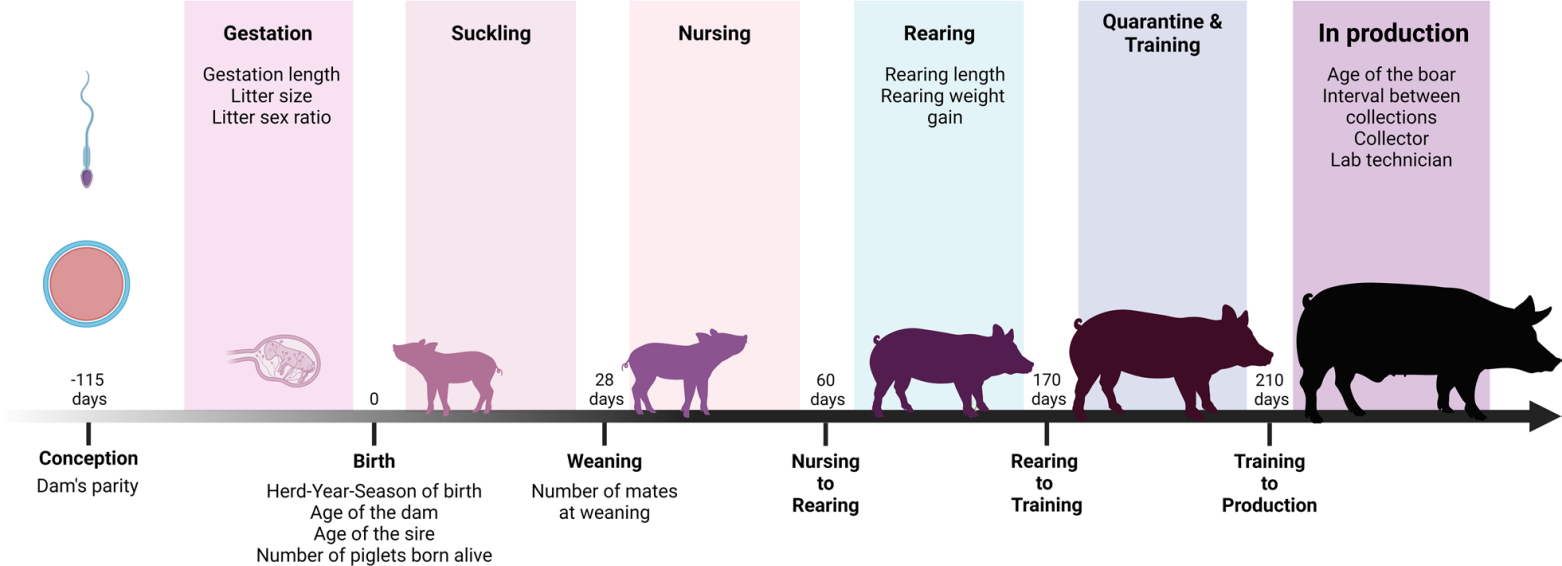
Representation of the life of an AI boar. AI boars undergo the following stages of development: (1) gestation, which lasts about 115 days after fecundation; (2) suckling, referring to the 28 days period after birth, in which piglets are milk-fed with the sow; (3) nursing, which is the first month after weaning, when pigs join other piglets for the first time in separate housing from the sows and depend entirely on a solid diet (no milk); (4) rearing, a period of strong growth for the boars, and (5) quarantine and training, in which boars undergo sanitary isolation and are trained to jump before going into semen production

A schematic representation of the life of an AI boar from conception to production, including the dam’s parity, is in Fig. 1. Conditions potentially affecting the boar’s subsequent semen traits during the boar’s gestation included gestation length, litter size, and litter sex ratio. Age of the dam and sire at birth of the boar and the number of piglets born alive in the boar’s litter were included as conditions experienced by the boar at birth. The number of litter mates at weaning was included as a weaning condition and length of the rearing period and weight gain during rearing were included as conditions describing the rearing period of the boars. A more detailed description of these effects is in Additional File 1, Table S1.

To test the significance of conception and early-life conditions, each term was added as a fixed class effect to model (1). Significance of the effect was based on the Wald-F test in ASREML v.4.2 [9]. Effects with a p-value < 0.05 (-log_10_(p-value) > 1.3) were considered suggestive. To account for multiple-testing (10 early-life conditions tested on 22 semen traits), the threshold was adjusted following a Bonferroni correction. Assuming 220 independent tests, effects with a p-value < 2.3 × 10^–4^ (-log_10_(p-value) > 3.6) were considered significant.

### Mitochondrial DNA effects

Normal sperm function has been linked to the activity of mitochondria [15]. To determine whether mitochondrial DNA explains variation in semen traits, a proxy for the mitochondrial type of a boar was created, as characteristics of mitochondria at the DNA level were not available. For this purpose, the mitochondrial inheritance of each boar was traced back in the pedigree following a similar approach as previously used in sheep [16] and bulls [17]. Assuming that mitochondrial DNA is exclusively transmitted through the maternal lineage, the mitochondrial DNA for each boar was traced back in the pedigree to the earliest known dam. Each of these founder dams was considered to have an unique mitochondrial DNA type and phenotypes of AI boars were assigned to these mitochondrial types. Considering only mitochondrial DNA types that were present in at least three boars with semen observations, a total of 85 different mitochondrial DNA types were established. All mitochondrial DNA types were traced back to a founder dam at least 10 generations back, with over 70% of mitochondrial DNA types traced to a founder dam at least 24 generations back. Mitochondrial DNA type was added as a random variable to model (1) and assumed to be distributed $\text{N}\left(0,\mathbf{I}{\upsigma }_{\text{mito}}^{2}\right)$, where **I** is the identity matrix and ${\upsigma }_{\text{mito}}^{2}$ is the mitochondrial DNA variance. The effects of mitochondrial DNA type and herd-year-season of birth were partially confounded because founder dams, and therefore mitochondrial DNA types, were exclusive to particular herds. Therefore, herd-year-season of birth was included as a fixed instead of a random effect (see model (1)) in these analyses in order to obtain a conservative estimate of mitochondrial DNA variance. Mitochondrial DNA effects were considered significant when 0 was not included in the 95% confidence interval of the estimated mitochondrial variance, which was computed as:

3 $${IC}_{95}=[\theta -1.96\cdot SE, \theta +1.96\cdot SE]$$

where θ is the estimated parameter and SE its standard error, as obtained from ASREML v4.2.

### Y chromosome effects

In mammals, several Y chromosome genes have male-specific functions [18]. In particular, semen quality and fertility traits have been linked to variation on the Y chromosome in mice [19] and bulls [20]. To estimate the effect of different Y chromosome haplotypes, inheritance of the Y chromosome was traced back in the pedigree following the strategy for mitochondrial inheritance but now reconstructing the inheritance through the paternal lineage. Y chromosome haplotypes were established based on the earliest known sire in the pedigree. Each of these founder sires was considered to have a unique Y chromosome haplotype. A total of 18 different Y chromosome haplotypes were established, which were each traced at least 25 generations back in the pedigree. The Y chromosome term was added to model (1) as a random variable and assumed to be distributed $\text{N}\left(0,\mathbf{I}{\upsigma }_{{\text{Y}}_{\text{chr}}}^{2}\right)$, where **I** is the identity matrix and ${\upsigma }_{{\text{Y}}_{\text{chr}}}^{2}$ is the Y chromosome variance. A Y chromosome effect was considered significant when the 95% confidence interval of the estimate of ${\upsigma }_{{\text{Y}}_{\text{chr}}}^{2}$, following Eq. (3), did not include 0.

### Maternal and paternal environment effects

Parental effects on semen traits have been observed in rabbits [21] and, more recently, in mice [22]. Maternal and paternal environment effects on boar semen production were evaluated. Here, we considered the maternal environmental effect to explain possible environmental conditions in the early-life of the boar’s dam, including systematic conditions during gestation, such as housing and feeding, and maternal behaviour and conditions during the 28-day period of suckling when piglets are fed on their mother’s milk and cohabitate with the dam. Similarly, the paternal environment effect was considered to explain environmental conditions experienced by the boar’s sire during sperm production that led to conception, such as housing and feeding conditions, that may impact semen characteristics of the AI boar.

For analyses investigating maternal and paternal effects, we only considered dams and sires with at least three offspring AI boars in the dataset. A total of 690 dams were considered, with 2837 offspring AI boars and 230,655 observations on semen traits. A total of 541 sires were included, with a total of 5336 offspring AI boars and 426,270 observations on semen traits. Total and progressive motility measured after one and two days of storage and abnormal acrosome were excluded from these analyses because of insufficient numbers of records. The maternal and paternal effects were evaluated in separate analyses by including maternal or paternal environment effects as random effects in model (1), assuming maternal environmental effects to be distributed $\text{N}\left(0,\mathbf{I}{\upsigma }_{{\text{e}}_{\text{m}}}^{2}\right)$, where **I** is the identity matrix and ${\upsigma }_{{\text{e}}_{\text{m}}}^{2}$ is the maternal environment variance, and paternal environmental effects $\text{N}\left(0,\mathbf{I}{\upsigma }_{{\text{e}}_{\text{p}}}^{2}\right)$, where **I** is the identity matrix and ${\upsigma }_{{\text{e}}_{\text{p}}}^{2}$ is the paternal environment variance. Due to partial confounding of these effects with herd-year-season of birth, the latter was included as a fixed effect in these analyses. Significance of maternal and paternal environment effects was determined based on a 95% confidence interval of the estimated variance components, following Eq. (3).

## Results

Descriptive statistics of the dataset used in this study are in Table 1. For semen quantity, semen volume displayed considerable variation, with an average of 391.0 ± 121.4 mL. Average sperm motility rate decreased after storage. For instance, the average rate of sperm cells with total motility was higher at 90.1% on fresh semen than after 1 day (82.9%), 2 days (85.0%), and 3 days (79.7%) of storage. Despite an overall lower mean motility with storage, we observed a slight increase in motility after 2 compared to 1 day of storage. This observation was unexpected and we could not identify a cause for this. We further investigated it by examining 343 commercial doses that were evaluated at 1, 2, and 3 days, which corroborated a decreasing motility trend with storage time: 85.9, 84.2, and 82.7%, respectively.

**Table 1:** Description of semen records of the boars

Trait^a^	Number of Boars	Number of observation	Mean	SD^b^	Min.^c^	Max.^d^
Semen Quantity						
Volume (mL)	5634	449,202	391.0	121.4	20	1010
Concentration (10^6^/mL)	5633	448,632	188.0	76.6	10	949
Total number of sperm cells (× 10^9^)	5634	449,361	70.4	26.9	0.01	226,4
Total number of normal sperm cells (× 10^9^)	5487	185,178	56.5	23.9	0.01	188,8
Total number of motile sperm cells (× 10^9^)	5634	449,372	63.0	24.9	0.01	211,0
Sperm Motility (%)						
Total motility of fresh semen	5599	425,885	90.1	4.8	3	100
Total motility after 1 day of storage	2864	45,659	82.9	8.8	20.3	99.5
Total motility after 2 days of storage	2888	48,939	85.0	7.7	21.9	99.6
Total motility after 3 days of storage	4784	190,319	79.7	10.6	0	99.8
Progressive motility of fresh semen	5631	445,745	80.9	8.2	0	99.5
Progressive motility after 1 day of storage	2868	45,739	73.3	9.8	3	97
Progressive motility after 2 days of storage	2894	49,035	75.0	9.1	10	98.1
Progressive motility after 3 days of storage	4785	190,533	70.9	11.4	0.3	99
Sperm Morphology (%)						
Total morphological abnormalities	5477	182,657	15.9	10.7	0	93
Total cytoplasmatic droplets	2737	186,206	8.8	5.5	0.4	63
Proximal cytoplasmatic droplets	2737	185,526	4.5	3.4	0	60.4
Distal cytoplasmatic droplets	2736	186,540	4.2	2.9	0	45.1
Distal midpiece reflex	2736	185,022	3.0	2.9	0	45.9
Coiled tail	2357	37,777	0.2	0.1	0	0.7
Bent tail	2734	172,224	0.8	0.7	0	10.5
Abnormal head	5229	116,941	1.0	1.6	0	40
Abnormal acrosome	1089	4,828	1.4	1.1	0	11

^a^All semen traits were measured with the CASA system, except abnormal head and abnormal acrosome which were measured using standard microscopy assessments. Semen traits of fresh semen were measured after pre-dilution

^b^*SD* Standard deviation of the trait

^c^*Min.* Minimum

^d^*Max.* Maximum

Total morphological abnormalities were present at an average rate of 15.9% (Table 1). Proximal and distal cytoplasmic droplets were the most prevalent individual morphological abnormalities, with a rate of 4.5 and 4.2%, respectively.

### Variance components, heritabilities, and repeatabilities

Estimates of genetic parameters for semen traits based on model (1) are in Table 2. In general, heritability estimates of semen traits were low to moderate (0.11–0.27) and repeatability estimates ranged from 0.20 to 0.65.

**Table 2:** Estimates of phenotypic and additive genetic variances, heritability (h^*2*^), repeatability (rep), and the percentage of variance explained by herd-year-season of birth (HYS, %) for semen traits

Trait	Phenotypic variance^a^	Additive genetic variance	h^2^	Rep	HYS (%)
Semen quantity					
Volume (mL)	11323	2441.7	0.19 _(0.02)_	0.41 _(0.01)_	2.6 _(0.5)_
Concentration (10^6^/mL)	4943.0	1271.5	0.19 _(0.02)_	0.45 _(0.01)_	2.7 _(0.5)_
Total number of sperm cells (10^9^)	589.66	112.5	0.21 _(0.02)_	0.40 _(0.01)_	0.7 _(0.2)_
Total number of normal sperm cells (10^9^)	477.79	83.6	0.21 _(0.02)_	0.39 _(0.01)_	0.5 _(0.2)_
Total number of motile sperm cells (10^9^)	515.98	91.0	0.21 _(0.01)_	0.39 _(0.01)_	0.7 _(0.3)_
Sperm motility					
Total motility of fresh semen	156.3	36.0	0.23 _(0.02)_	0.58 _(0.01)_	2.8 _(0.6)_
Total motility after 1 day of storage	202.2	43.9	0.22 _(0.02)_	0.35 _(0.01)_	0.9 _(0.4)_
Total motility after 2 days of storage	217.8	48.2	0.22 _(0.02)_	0.36 _(0.01)_	5.1 _(1.1)_
Total motility after 3 days of storage	298.5	71.1	0.24 _(0.02)_	0.40 _(0.01)_	2.3 _(0.5)_
Progressive motility of fresh semen	242.4	57.6	0.24 _(0.02)_	0.50 _(0.01)_	3.4 _(0.6)_
Progressive motility after 1 day of storage	186.2	39.8	0.21 _(0.02)_	0.35 _(0.01)_	1.7 _(0.6)_
Progressive motility after 2 days of storage	194.3	39.6	0.20 _(0.02)_	0.35 _(0.01)_	2.4 _(0.7)_
Progressive motility after 3 days of storage	232.7	53.4	0.23 _(0.02)_	0.41 _(0.01)_	1.8 _(0.4)_
Sperm morphology					
Total morphological abnormalities	4261.7	886.7	0.21 _(0.02)_	0.52 _(0.01)_	2.9 _(0.6)_
Total cytoplasmic droplets	3135.9	708.6	0.23 _(0.03)_	0.55 _(0.01)_	8.0 _(1.8)_
Proximal cytoplasmic droplets	3408.0	825.9	0.24 _(0.03)_	0.56 _(0.01)_	7.9 _(1.8)_
Distal cytoplasmic droplets	2605.6	641.4	0.25 _(0.03)_	0.51 _(0.01)_	4.0 _(1.1)_
Distal midpiece reflex	3563.5	957.7	0.27 _(0.03)_	0.65 _(0.01)_	1.3 _(0.6)_
Coiled tail	31.7	3.9	0.12 _(0.02)_	0.28 _(0.01)_	1.2 _(0.4)_
Bent tail	985.8	112.4	0.11 _(0.02)_	0.28 _(0.01)_	1.0 _(0.4)_
Abnormal head	3369.6	399.4	0.12 _(0.01)_	0.23 _(0.01)_	0.8 _(0.2)_
Abnormal acrosome	771.8	110.7	0.14 _(0.04)_	0.20 _(0.02)_	5.0 _(1.5)_

All semen traits were measured with CASA systems, except abnormal head and abnormal acrosome which were measured using standard microscopy assessments

^a^Phenotypic variance was calculated based on the sum of additive genetic, permanent environment, herd-year-season of birth and residual variances. Standard errors are shown in subscript and were < 0.05 for heritability and < 0.03 for repeatability

Parameters were estimates for untransformed semen quantity and transformed sperm motility and sperm morphology traits

Herd-year-season of birth accounted for a significant proportion of variance in semen traits, ranging from 0.5 to 8.0% of phenotypic variance (Table 2). The highest estimates of herd-year-season of birth effects were for total motility after 2 days of storage (5.1%), proximal cytoplasmic droplets (7.9%), and total cytoplasmic droplets (8%), while the lowest estimates were for total number of normal sperm cells (0.5%), total number of cells, and total number of motile sperm cells (0.7%).

Estimates of heritabilities for semen quantity traits ranged from 0.19 to 0.21 (Table 2). Repeatability estimates ranged from 0.39 for total number of motile and normal sperm cells to 0.45 for ejaculate concentration.

Heritability estimates for motility traits of fresh semen were comparable to those observed after storage, ranging from 0.20 to 0.24 (Table 2). Repeatability estimates after storage were lower than for fresh semen: 0.58 for total motility of fresh semen versus 0.40 for total motility after three days of storage and 0.50 for progressive motility of fresh semen versus 0.41 for progressive motility after three days of storage.

Estimates of heritabilities for morphology traits based on CASA were low to moderate, ranging from 0.11 to 0.27 (Table 2), while estimates of repeatability ranged from 0.28 to 0.65. Estimates of heritability for morphology traits measured with microscopy (i.e. abnormal head and abnormal acrosome) were between 0.12 and 0.14, while estimates of repeatability ranged from 0.20 to 0.23.

Estimates for sperm motility and morphology traits were based on transformed traits; estimates on untransformed traits using the same base model are in Additional File 2, Table S2. Estimates of repeatability for motility and morphology traits changed substantially after transformation whereas heritability estimates showed minor changes after transformation. For example, the estimate of repeatability for untransformed total motility of fresh semen was 0.71 and decreased to 0.58 for the transformed trait (Table 2).

### Genetic and phenotypic correlations

**Fig. 2:**
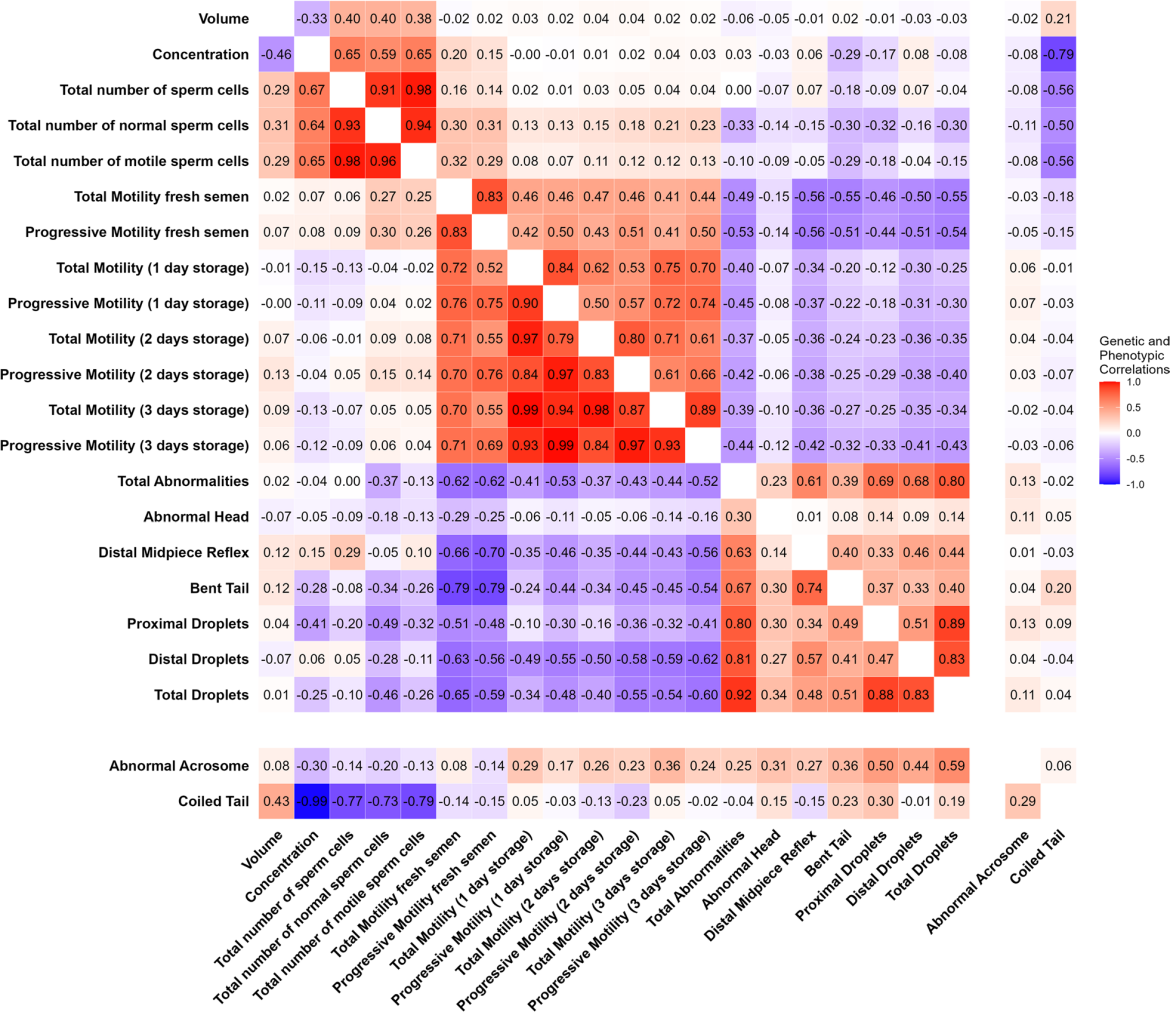
Phenotypic and genetic correlations between semen traits. Phenotypic correlations above the diagonal and genetic correlations below the diagonal

Estimates of genetic and phenotypic correlations are represented in Fig. 2. All estimated correlations and their standard errors, including permanent environmental, herd-year-season of birth, and residual correlations can be found in Additional File 3, Table S3.

In general, high positive genetic and phenotypic correlations were estimated among semen quantity traits, sperm motility traits, and sperm morphology traits (Fig. 2). Weak genetic correlations were estimated between semen quantity and semen quality traits, which include both motility and morphology traits.

For semen quantity traits, a moderate negative genetic correlation was estimated between ejaculate concentration and volume (− 0.46 ± 0.05) (Fig. 2). High genetic correlations were estimated between total number of sperm cells, total number of normal sperm cells, and total number of motile sperm cells, ranging from 0.93 ± 0.01 to 0.98 ± 0.01.

For motility traits, genetic correlations for total and progressive motility after storage were estimated to be high and positive, ranging from 0.79 ± 0.04 to 0.99 ± 0.01 (Fig. 2). However, estimates of genetic correlations between motility traits of fresh semen and after storage were lower, ranging from 0.52 ± 0.05 between progressive motility of fresh semen and total motility after one day of storage to 0.76 ± 0.03 between progressive motility of fresh semen and after two days of storage.

For sperm morphology traits, the estimated genetic and phenotypic correlations between coiled tail and abnormal acrosome and other semen traits showed very different patterns (Fig. 2). Aside from these traits, estimates of genetic correlations ranged from 0.14 ± 0.09 for abnormal head and distal midpiece reflex to 0.92 ± 0.02 between total morphological abnormalities and total cytoplasmic droplets.

Both genetic and phenotypic correlations between motility and morphology traits were estimated to be moderately negative, with estimates of the genetic correlation ranging from − 0.05 ± 0.07 between abnormal head and total motility after 2 days of storage to − 0.79 ± 0.04 between bent tail and total and progressive motility of fresh semen (Fig. 2).

### Early-life conditions

Results for log-transformed p-values for early-life effects on semen traits are shown in Additional File 4, Table S4. Overall, no early-life effect passed the significance threshold (i.e. after adjusting for multiple testing), but several showed suggestive effects (p < 0.05). The effect of age of the dam was suggestive for morphology traits, namely on total (p-value = 0.001) and proximal (p-value = 0.02) cytoplasmic droplets, while age of the sire was suggestive for semen quantity traits, particularly on ejaculate volume (p-value = 0.004) and concentration (p-value = 0.04). For effects at conception, parity of the dam exhibited a suggestive effect (p-value = 0.03) on distal midpiece reflex. Among early-life effects, gestation length had a suggestive effect on progressive motility after two days of storage (p-value = 0.03) and on abnormal acrosome (p-value = 0.008). Litter size showed a suggestive effect on total (p-value = 0.03) and progressive motility after three days of storage (p-value = 0.03) and on total morphological abnormalities (p-value = 0.04). Neither the sex ratio of the litter that the AI boar was born in, nor the number of mates at weaning had significant or suggestive effects on semen traits.

### Effects of mitochondria and the Y chromosome

Estimated percentages of the phenotypic variance explained by mitochondrial DNA and the Y chromosome are shown in Additional File 5, Table S5. The largest estimates of mitochondrial DNA variance were observed for total and progressive motility after two days of storage, at 0.9 and 1.0% of the phenotypic variance, respectively, but they did not significantly deviate from 0. The largest estimate for variance explained by the Y chromosome was for semen concentration, representing 3.6% of the phenotypic variance in the trait but this also did not significantly deviate from 0.

### Maternal and paternal environment effects

The percentages of phenotypic variation explained by maternal and paternal environment variances are in Table 3. For cases where a significant part of the variation was explained by maternal or paternal environmental effects, resulting heritability and repeatability estimates were also reported in Table 3. In general, a maternal environment effect was detected for motility traits, while a paternal environment effect was reported for a small number of sperm quality traits, but they did not exceed 1.5% of the phenotypic variance. Maternal environment effects for motility traits ranged from 2.3% of phenotypic variance for progressive motility after three days of storage to 4.6% for total motility of fresh semen. A maternal effect of 3.1 and 1.5% of the phenotypic variance was estimated for total morphological abnormalities and ejaculate volume, respectively.

**Table 3:** Estimates of the percentage of phenotypic variance explained by maternal and paternal environment effects, and of heritabilities and repeatabilities for the corresponding models

Trait	Maternal variance (%)	h^2^	Rep	Paternal variance (%)	h^2^	Rep
Semen Quantity						
Volume (mL)	1.5 _(0.9)_	0.22 _(0.03)_	0.37 _(0.01)_	0.6 _(0.4)_	–	–
Concentration (10^6^/mL)	< 0.01	–	–	0.4 _(0.4)_	–	–
Total number of sperm cells (10^9^)	0.9 _(0.8)_	–	–	0.7 _(0.4)_	–	–
Total number of normal sperm cells (10^9^)	1.0 _(0.9)_	–	–	0.7 _(0.5)_	–	–
Total number of motile sperm cells (10^9^)	1.1 _(0.8)_	–	–	0.7 _(0.4)_	–	–
Sperm Motility^a^						
Total motility of fresh semen	4.6 _(1.4)_	0.14 _(0.03)_	0.53 _(0.01)_	0.5 _(0.6)_	–	–
Total motility after 3 days of storage	2.5 _(1.1)_	0.15 _(0.03)_	0.37 _(0.02)_	1.3 _(0.5)_	0.20 _(0.02)_	0.39 _(0.02)_
Progressive motility of fresh semen	2.8 _(1.2)_	0.16 _(0.03)_	0.47 _(0.02)_	0.4 _(0.5)_	–	–
Progressive motility after 3 days of storage	2.3 _(1.1)_	0.15 _(0.03)_	0.39 _(0.02)_	1.2 _(0.5)_	0.21 _(0.02)_	0.40 _(0.02)_
Sperm Morphology^a^						
Total morphological abnormalities	3.1 _(1.2)_	0.12 _(0.03)_	0.49 _(0.02)_	1.5 _(0.6)_	0.18 _(0.02)_	0.52 _(0.02)_
Total cytoplasmatic droplets	2.6 _(1.8)_	–	–	0.9 _(0.9)_	–	–
Proximal cytoplasmatic droplets	2.9 _(1.9)_	–	–	0.1 _(0.8)_	–	–
Distal cytoplasmatic droplets	1.0 _(1.6)_	–	–	0.8 _(0.8)_	–	–
Distal midpiece reflex	2.9 _(2.0)_	–	–	1.5 _(1.0)_	–	–
Coiled tail	< 0.01	–	–	0.2 _(0.5)_	–	–
Bent tail	0.7 _(0.9)_	–	–	< 0.01	–	–
Abnormal head	0.7 _(0.6)_	–	–	0.2 _(0.3)_	–	–

Heritability and repeatability estimates for which maternal and paternal variances did not significantly deviate from zero were omitted. Standard errors are in subscript

^a^Maternal and paternal environment variances were estimates for transformed trait values

Among traits demonstrating a significant maternal environment effect, a large decrease in heritability and a small decrease in repeatability estimates were observed when accounting for this effect (Table 3) as compared to the estimates using the base repeatability model (Table 2), except for ejaculate volume. The heritability estimate for total morphological abnormalities decreased from 0.21 to 0.12. For motility traits, heritability estimates decreased from 0.23 to 0.24 with the base model to 0.14–0.16 with the maternal effects model.

A paternal environment effect was estimated for total and progressive motility after three days of storage (Table 3), as well as total morphological abnormalities, accounting for 1.3, 1.2, and 1.5% of the phenotypic variance, respectively. Heritability and repeatability estimates for these traits showed a small decrease when including the paternal environment effect in the model.

## Discussion

In this study, we provide a comprehensive analysis of genetic and systematic environmental effects on both common and novel porcine semen traits. Our analysis is based on a large dataset of semen records collected during routine assessments of semen quality in a purebred pig line. This dataset includes extensive pedigree information, as well as documented factors from multiplication farms before boars are introduced to AI stations. The comprehensive pedigree allowed us to explore non-autosomal inheritance and parental environmental effects not previously studied on porcine semen traits. By examining documentation from multiplication farms, we also addressed the impact of early-life conditions on later semen production. We provided estimates of genetic parameters for semen traits, including morphology traits assessed using CASA and microscopy, and provided new estimates for sperm motility traits after storage. We identified a significant maternal environment effect, particularly for sperm motility traits, and incorporated a maternal environment term into the model to refine genetic parameter estimates. Our investigation into mitochondrial DNA and Y chromosome inheritances revealed no discernible effects on semen traits. We also report suggestive effects of conception and early-life conditions on semen production later in life but negligible paternal environmental effects.

### Heritability and repeatability

Genetic parameters were estimated for both transformed and untransformed semen traits. Estimates of heritabilities remained moderate and stable, while repeatability estimates decreased substantially after transformation (for estimates on transformed traits, see Table 2; for estimates on untransformed traits, see Additional File 2, Table S2). To our knowledge, estimates for transformed semen quality traits have not been reported previously. However, our estimates of low to moderate estimates of heritability (0.12–0.27) and of repeatability (0.39–0.65) after transformation were similar to those previously reported for untransformed traits [11, 23–25]. Nevertheless, our observations indicate that repeatability estimates are sensitive to skewness.

Among heritabilities and repeatabilities estimated, we report genetic parameters for sperm abnormality traits measured with CASA systems and with microscopy assessment (i.e., abnormal head and abnormal acrosome). Sperm abnormality traits measured with CASA had low to moderate heritability estimates (0.11–0.27), while traits measured with microscopy had lower estimates (0.12–0.14). These estimates are consistent with literature, both in the case of moderate heritabilities for CASA traits [11, 23] and lower heritabilities for semen traits measured with microscopy [26]. It is worth noting that a low correlation has been observed for morphology traits measured with a CASA system and microscopy [27]. We also noted that the effect of the lab technicians responsible for measuring these traits was two to three times larger for morphology traits measured with microscopy as compared to CASA. This indicates that subjectivity introduced by microscopy assessment may result in noise, leading to lower heritability estimates.

We also estimated genetic parameters for sperm motility traits after storage, which to the best of our knowledge has not been done previously. Heritability estimates of motility after storage (0.20–0.24) were moderate and consistent with heritability estimates of fresh semen motilities (0.23–0.24). However, repeatability estimates for stored semen motilities (0.35–0.40) were lower than those found for fresh semen (0.50–0.58). Our estimates for fresh semen traits are consistent with those reported in the literature [23]. These observations indicate that storage of semen after collection reduces the correlation between repeated motility records due to a smaller permanent environment effect. This has implications when selective breeding strategies include semen traits. It is noteworthy that with lower repeatability, there is less certainty about the sperm motility of future ejaculates of the same boar. Therefore, it is important to have a continued assessment of sperm motility after storage of all ejaculates.

We also reported estimates of heritability and repeatability after accounting for a maternal environmental effect. By including this effect, heritability estimates substantially decreased (to 0.12–0.16), while repeatability estimates decreased slightly as compared to a model without a maternal environmental effect. In livestock species, reproductive traits tend to have low heritabilities [28]. However, in the particular case of semen traits in pigs, moderate heritability estimates (0.20–0.30) have been systematically reported in literature [11, 24, 25]. It is worth noting that maternal environmental effects have not been reported previously when estimating genetic parameters of semen traits. This indicates that disregarding maternal environmental effects may result in an overestimation of the heritability of sperm motility traits. This has been pointed out for other traits in chickens and sheep as well [29, 30]. Thus, when possible, maternal environmental effects should be accounted for when analysing semen traits. To enable modelling maternal environmental effects, data on a minimum of three offspring boars per dam should be present in the dataset, which may pose a limitation when most AI boars are offspring from different dams. In commercial populations, this limitation is mostly due to the small number of boars that qualify for AI, which often leads to the absence of siblings in the dataset.

### Genetic and phenotypic correlations

Our results indicate that phenotypic and genetic correlations were higher between traits of the same trait group, i.e., semen quantity, sperm motility, and sperm morphology (Fig. 2). Similar observations have been made for semen traits in other pig populations [11, 23, 26]. A focal point in this analysis was the genetic correlations between fresh and stored sperm motility traits. Estimates of genetic correlations were notably lower (0.52–0.76) between motility traits of fresh and stored semen than between motility traits of semen stored for different time periods (0.79–0.99). This indicates that motilities of fresh and stored semen are genetically different traits. Currently, motility assessed on fresh semen is an important criterion to assess the quality of the ejaculate. However, semen for commercial use can be stored and used for insemination several days after collection. Our results emphasize the importance of also considering sperm motility after storage. Recent publications have described differences between fresh and stored boar semen, including a relationship between semen storage and lower mitochondrial activity [31, 32], increased DNA damage [33] and higher apoptosis rate [34]. Future research should consider the genetic differences between motility of fresh and stored semen that were highlighted in this study and assess the implications of motility after storage on fertility outcomes.

### Mitochondrial DNA and Y chromosome

We also addressed whether non autosomal inheritance could explain variation in semen traits. We considered mitochondrial DNA and Y chromosome as exclusively maternal and paternal inheritances, respectively, and traced them through the pedigree (for estimates, see Additional File 5, Table S5). These analyses revealed no discernible effects of mitochondrial DNA or the Y chromosome on semen quality traits. These findings suggest that, within the studied population, variation in mitochondrial DNA or Y chromosome does not contribute significantly to the observed variation in semen traits. Similar observations have been made when estimating the effect of mitochondrial DNA variation on semen traits in bulls with a similar approach [17]. However, this strategy has not been previously used to identify contributions of the Y chromosome to semen traits. It is possible that a pedigree-based approach does not have sufficient resolution to detect relevant variation in mitochondrial DNA and the Y chromosome or the variation in mitochondrial DNA and Y chromosome haplotypes might be limited in this population. In this regard, it is worth noting that many boars carry the same Y chromosome, as nearly 70% of all boars were represented by two Y chromosomes.

### Systematic environment affecting semen production

It has been suggested that conditions of the litter-of-origin may influence reproductive performance of pigs later in life [35]. A focal point in this study was to examine whether environmental effects of the parents and systematic conditions at conception and during early-life of the boar had permanent effects on later semen production. With regards to parental environment effects, our findings indicated relevant maternal environmental effects, predominantly on motility traits, and negligible paternal effects (Table 3). To our knowledge, no previous study has considered maternal or paternal environment effects on offspring semen traits. However, maternal effects have been considered for meat production and female reproductive traits in cattle [36, 37] and for production traits in chicken [38, 39], emphasizing how disregarding these effects can lead to overestimated heritabilities. In our case, a maternal term was added to the base model, including only dams with at least three offspring boars. Most dams had offspring from multiple parities, with most parities having one or two offspring boars in the dataset. Due to this structure, a litter-of-origin effect could not be addressed but it was likely confounded with the maternal effects term. Furthermore, this maternal effects term can also explain maternal temporary environmental effects that are specific to each parity, i.e., age or parity of the dam. In this population, boars experience a standardized postnatal environment, involving a 28-day suckling period with the dam. Thus the maternal effect may be related to maternal behaviour and nutrition conditions during gestation, including gestation length, suckling conditions, and the number of litter mates born alive. In this regard, development of the testes and release of reproductive hormones have been documented during gestation, with ongoing developmental processes extending beyond birth [40], which overlaps the period in which maternal care is most important. Further studies should focus on pinpointing the origin of these maternal environment effects on subsequent semen traits of the male progeny. Unlike sows, sires do not actively contribute during the gestation period of their piglets, nor do they share close quarters with them during neonatal development. This paternal separation may explain the negligible paternal effects observed on semen traits.

With regards to conditions at conception and during early-life, the effects considered displayed suggestive or no impact on semen traits in this population. To our knowledge, attempts to disentangle these effects on semen traits have not been made previously. These observations indicate that the early-life conditions addressed in this study do not contribute to semen traits in this population, which may be attributed to the standardization of early-life of the boars in commercial pig production systems. Among early-life conditions, herd-year-season of birth merited special attention, as our results indicate that it accounted for 0.5–8% of phenotypic variance in semen traits (Table 2). The effect of herd-year-season of birth was, however, partially confounded with other early-life conditions. Nevertheless, the substantial variance accounted for by herd-year-season of birth indicates that it should be considered while analysing semen traits.

## Conclusions

The current study represents a step towards unravelling the influence of parental and early-life conditions on boar semen production. When addressing porcine semen traits, we recommend the assessment of normality of semen traits and highlight the importance of assessing sperm motility also after storage. We concluded that sperm motility traits measured on fresh semen and after storage are genetically different traits. Early-life conditions can have an effect on later semen quantity and quality traits. We did not detect significant effects of mitochondrial DNA and the Y chromosome on semen traits. Maternal environmental effects significantly contributed to variation in some semen traits and including this term in the model affected genetic parameter estimates. These insights highlight the intricate interplay of systematic factors that shape boar semen quality.

## Supplementary Information

Additional file 1: Table S1 Descriptive statistics of early-life fixed effects.

Additional file 2: Table S2 Phenotypic and additive genetic variances, heritability (h^2^), repeatability (rep) and percentage of variance for herd-year-season of birth for the untransformed traits of sperm motility and morphology.

Additional file 3: Table S3 Phenotypic, genetic, permanent environment, herd-year-season of birth n and residual correlations and respective standard errors, between semen traits.

Additional file 4: Table S4 Significance of early-life conditions on semen production traits.

Additional file 5: Table S5 Percentage of the phenotypic variance that can be explained by Mitochondria and Y chromosome.

## Data Availability

The dataset analysed in this study is property of Topigs Norsvin Research Center B.V. The dataset is available from Barbara Harlizius (barbara.harlizius@topigsnorsvin.com) upon reasonable request.
