# Supplementary material for: Genetic parameters and parental and early-life effects of boar semen traits
Source: Genet Sel Evol. 2025 Feb 6;57:4. doi: 10.1186/s12711-025-00954-6 (PMC11800458; doi:10.1186/s12711-025-00954-6)
Supplement: Supplementary file 1 — Additional file 1: Table S1 Descriptive statistics of early-life fixed effects. [file 12711_2025_954_MOESM1_ESM.docx]

**Descriptive statistics of early-life fixed effects.**

| **Effect** | **Fit** | **Description** | **No. of boars^a)^** | **No. of classes^a)^** | **Min.^b)^ – Max.^c)^** |
| --- | --- | --- | --- | --- | --- |
| Age of the dam | Categorical | Age of the dam at birth (in months). | 5,692 | 17 | 10-31 |
| Age of the sire | Categorical | Age of the sire at birth (in months). | 5,692 | 27 | 10-42 |
| Dam parity | Categorical | Number of times the boar’s dam had offspring. | 4,854 | 6 | 1-6 |
| Gestation length | Categorical | Duration of the gestation period (in days). | 4,854 | 9 | 112-120 |
| Litter size | Categorical | Total number of born piglets in the boar’s litter. | 4,854 | 12 | 5-16 |
| Litter sex ratio | Categorical | Ratio of piglets over gilts in the boar’s litter. | 2,597 | 12 | 0.44-5 |
| Mates at weaning | Categorical | Number of litter mates during weaning. | 4,854 | 11 | 4-14 |
| Number of born alive | Categorical | Number of litter mates born alive. | 4,854 | 11 | 5-15 |
| Rearing length | Categorical | Duration of the rearing period (in days). | 4,740 | 33 | 60-107 |
| Rearing weight growth | Categorical | Weight growth during rearing (in kilograms). | 4,740 | 29 | 70-114 |

^a)^No=Number.

^b)^Min =Minimum

^c)^Max=Maximum.
