# Supplementary material for: Genetic parameters and parental and early-life effects of boar semen traits
Source: Genet Sel Evol. 2025 Feb 6;57:4. doi: 10.1186/s12711-025-00954-6 (PMC11800458; doi:10.1186/s12711-025-00954-6)
Supplement: Supplementary file 2 — Additional file 2: Table S2 Phenotypic and additive genetic variances, heritability (h2), repeatability (rep) and percentage of variance for herd-year-season of birth for the untransformed traits of sperm motility and morphology. [file 12711_2025_954_MOESM2_ESM.docx]

**Phenotypic and additive genetic variances, heritability (h*^2^*), repeatability (rep) and percentage of variance for herd-year-season of birth for the untransformed traits of sperm motility and morphology.**

| **Trait** | **Phenotypic**  **variance ^a)^** | **Additive genetic variance** | | ***h^2^*** | | ***rep*** | | **HYS (%)** | |
| --- | --- | --- | --- | --- | --- | --- | --- | --- | --- |
| **Sperm Motility (%)** | | | | | | | | | |
| Total motility of fresh semen | 43.9 | | 11.8 | | 0.27 _(0.03)_ | | 0.71 _(0.01)_ | | 2.3 _(0.6)_ |
| Total motility after 1 day of storage | 59.1 | | 13.7 | | 0.23 _(0.02)_ | | 0.37 _(0.01)_ | | 1.2 _(0.5)_ |
| Total motility after 2 days of storage | 58.1 | | 13.9 | | 0.24 _(0.02)_ | | 0.39 _(0.01)_ | | 5.0 _(1.1)_ |
| Total motility after 3 days of storage | 115.5 | | 27.7 | | 0.24 _(0.02)_ | | 0.45 _(0.01)_ | | 2.3 _(0.5)_ |
| Progressive motility of fresh semen | 108.2 | | 23.5 | | 0.22 _(0.02)_ | | 0.63 _(0.01)_ | | 2.9 _(0.6)_ |
| Progressive motility after 1 day of storage | 86.3 | | 19.0 | | 0.22 _(0.02)_ | | 0.36 _(0.01)_ | | 2.3 _(0.7)_ |
| Progressive motility after 2 days of storage | 81.8 | | 17.8 | | 0.22 _(0.02)_ | | 0.37 _(0.01)_ | | 2.5 _(0.7)_ |
| Progressive motility after 3 days of storage | 141.2 | | 32.8 | | 0.23 _(0.02)_ | | 0.45 _(0.01)_ | | 2.1 _(0.5)_ |
| **Sperm Morphology (%)** | | | | | | | | | |
| Total morphological abnormalities | 144.9 | | 25.8 | | 0.18 _(0.02)_ | | 0.53 _(0.01)_ | | 2.8 _(0.6)_ |
| Total Cytoplasmic droplets | 45.4 | | 8.6 | | 0.19 _(0.03)_ | | 0.60 _(0.01)_ | | 6.3 _(1.5)_ |
| Proximal Cytoplasmic droplets | 19.4 | | 3.8 | | 0.20 _(0.03)_ | | 0.66 _(0.01)_ | | 5.5 _(1.4)_ |
| Distal Cytoplasmic droplets | 10.8 | | 2.3 | | 0.21 _(0.03)_ | | 0.56 _(0.01)_ | | 3.5 _(1.0)_ |
| Distal Midpiece Reflex | 13.5 | | 2.5 | | 0.18 _(0.03)_ | | 0.72 _(0.01)_ | | 0.9 _(0.5)_ |
| Coiled Tail | < 0.01 | < 0.01 | | 0.12 _(0.02)_ | | 0.29 _(0.01)_ | | 1.2 _(0.4)_ | |
| Bent Tail | 0.5 | 0.1 | | 0.11 _(0.02)_ | | 0.37 _(0.01)_ | | 0.7 _(0.4)_ | |
| Abnormal Head | 2.7 | 0.4 | | 0.14 _(0.01)_ | | 0.37 _(0.01)_ | | 1.5 _(0.4)_ | |
| Abnormal Acrosome | 0.6 | 0.1 | | 0.12 _(0.04)_ | | 0.19 _(0.02)_ | | 2.1 _(1.0)_ | |

Standard errors are shown in subscript and were < 0.05 for heritability and <0.03 for repeatability.

1. Phenotypic variance was calculated based on the sum of additive genetic, permanent environment, herd-year-season of birth and residual variances.
