# Supplementary material for: Genetic parameters and parental and early-life effects of boar semen traits
Source: Genet Sel Evol. 2025 Feb 6;57:4. doi: 10.1186/s12711-025-00954-6 (PMC11800458; doi:10.1186/s12711-025-00954-6)
Supplement: Supplementary file 3 — Additional file 3: Table S3 Phenotypic, genetic, permanent environment, herd-year-season of birth n and residual correlations and respective standard errors, between semen traits. [file 12711_2025_954_MOESM3_ESM.docx]

**Phenotypic, genetic, permanent environment, herd-year-season of birth and residual correlations and respective standard errors, between semen traits.**

| **Trait 1** | **Trait 2** | ***r_p_*** | ***r_a_*** | ***r_pe_*** | ***r_hys_*** | ***r_e_*** |
| --- | --- | --- | --- | --- | --- | --- |
| Volume | Concentration | -0.33 (0.01) | -0.46 (0.05) | -0.17 (0.04) | -0.81 (0.07) | -0.30 (<0.01) |
| Volume | Total number of sperm cells | 0.40 (0.01) | 0.29 (0.05) | 0.54 (0.03) | 0.25 (0.17) | 0.40 (<0.01) |
| Volume | Total number of normal sperm cells | 0.40 (0.01) | 0.31 (0.06) | 0.53 (0.03) | 0.13 (0.21) | 0.40 (<0.01) |
| Volume | Total number of motile sperm cells | 0.38 (0.01) | 0.29 (0.05) | 0.50 (0.03) | 0.14 (0.18) | 0.38 (<0.01) |
| Volume | Coiled Tail | 0.21 (0.01) | 0.43 (0.08) | 0.01 (0.06) | 0.24 (0.23) | 0.20 (0.01) |
| Volume | Bent Tail | 0.02 (0.01) | 0.12 (0.09) | -0.07 (0.05) | 0.18 (0.24) | 0.02 (<0.01) |
| Volume | Distal Midpiece Reflex | -0.01 (0.01) | 0.12 (0.09) | -0.10 (0.05) | -0.49 (0.24) | -0.01 (<0.01) |
| Volume | Proximal cytoplasmatic droplets | -0.01 (0.02) | 0.04 (0.09) | -0.14 (0.05) | 0.54 (0.14) | -0.04 (<0.01) |
| Volume | Distal cytoplasmatic droplets | -0.03 (0.01) | -0.07 (0.09) | -0.04 (0.06) | 0.33 (0.19) | -0.04 (<0.01) |
| Volume | Total cytoplasmatic droplets | -0.03 (0.02) | 0.01 (0.09) | -0.13 (0.05) | 0.48 (0.15) | -0.05 (<0.01) |
| Volume | Abnormal Head | -0.05 (0.01) | -0.07 (0.06) | -0.13 (0.04) | -0.15 (0.17) | -0.02 (<0.01) |
| Volume | Abnormal Acrosome | -0.02 (0.02) | 0.08 (0.12) | -0.13 (0.15) | 0.04 (0.18) | -0.03 (0.02) |
| Volume | Total morphological abnormalities | -0.06 (0.01) | 0.02 (0.07) | -0.18 (0.04) | 0.14 (0.14) | -0.05 (<0.01) |
| Volume | Total motility of fresh semen | -0.02 (0.01) | 0.02 (0.07) | 0.01 (0.04) | -0.28 (0.14) | -0.04 (<0.01) |
| Volume | Progressive motility of fresh semen | 0.02 (0.01) | 0.07 (0.06) | 0.03 (0.04) | -0.08 (0.14) | -0.01 (<0.01) |
| Volume | Total motility after one day of storage | 0.03 (0.01) | -0.01 (0.07) | -0.01 (0.06) | 0.92 (0.12) | 0.02 (<0.01) |
| Volume | Progressive motility after one day of storage | 0.02 (0.01) | 0.01 (0.07) | -0.03 (0.06) | 0.82 (0.12) | 0.01 (<0.01) |
| Volume | Total motility after two days of storage | 0.04 (0.01) | 0.07 (0.07) | -0.08 (0.06) | 0.65 (0.10) | 0.02 (<0.01) |
| Volume | Progressive motility after two days of storage | 0.04 (0.01) | 0.13 (0.07) | -0.11 (0.06) | 0.87 (0.09) | 0.01 (<0.01) |
| Volume | Total motility after three days of storage | 0.02 (0.01) | 0.09 (0.06) | 0.05 (0.05) | 0.39 (0.13) | -0.03 (<0.01) |
| Volume | Progressive motility after three days of storage | 0.02 (0.01) | 0.06 (0.06) | 0.04 (0.05) | 0.45 (0.14) | -0.02 (<0.01) |
| Concentration | Total number of sperm cells | 0.65 (<0.01) | 0.67 (0.03) | 0.66 (0.02) | 0.19 (0.18) | 0.66 (<0.01) |
| Concentration | Total number of normal sperm cells | 0.59 (0.01) | 0.64 (0.04) | 0.57 (0.03) | 0.27 (0.20) | 0.60 (<0.01) |
| Concentration | Total number of motile sperm cells | 0.65 (<0.01) | 0.65 (0.03) | 0.67 (0.02) | 0.34 (0.16) | 0.66 (<0.01) |
| Concentration | Coiled Tail | -0.79 (<0.01) | -0.99 (<0.01) | -0.95 (0.01) | -0.97 (0.01) | -0.64 (<0.01) |
| Concentration | Bent Tail | -0.29 (0.01) | -0.28 (0.09) | -0.48 (0.05) | -0.80 (0.11) | -0.22 (<0.01) |
| Concentration | Distal Midpiece Reflex | 0.06 (0.02) | 0.15 (0.09) | 0.09 (0.06) | -0.18 (0.30) | 0.01 (<0.01) |
| Concentration | Proximal cytoplasmatic droplets | -0.17 (0.02) | -0.41 (0.08) | -0.28 (0.06) | 0.16 (0.22) | 0.01 (<0.01) |
| Concentration | Distal cytoplasmatic droplets | 0.08 (0.02) | 0.06 (0.09) | -0.02 (0.07) | 0.12 (0.23) | 0.13 (<0.01) |
| Concentration | Total cytoplasmatic droplets | -0.08 (0.02) | -0.25 (0.09) | -0.21 (0.06) | 0.07 (0.22) | 0.06 (<0.01) |
| Concentration | Abnormal Head | -0.03 (0.01) | -0.05 (0.06) | -0.07 (0.05) | 0.01 (0.17) | -0.02 (<0.01) |
| Concentration | Abnormal Acrosome | -0.08 (0.02) | -0.30 (0.11) | -0.10 (0.13) | -0.21 (0.18) | -0.01 (0.02) |
| Concentration | Total morphological abnormalities | 0.03 (0.01) | -0.04 (0.06) | 0.04 (0.04) | -0.07 (0.15) | 0.06 (<0.01) |
| Concentration | Total motility of fresh semen | 0.20 (0.01) | 0.07 (0.06) | 0.33 (0.04) | 0.23 (0.14) | 0.20 (<0.01) |
| Concentration | Progressive motility of fresh semen | 0.15 (0.01) | 0.08 (0.06) | 0.31 (0.04) | 0.12 (0.14) | 0.12 (<0.01) |
| Concentration | Total motility after one day of storage | 0.01 (0.01) | -0.15 (0.07) | 0.30 (0.06) | -0.57 (0.19) | -0.02 (<0.01) |
| Concentration | Progressive motility after one day of storage | -0.01 (0.01) | -0.11 (0.07) | 0.21 (0.06) | -0.57 (0.15) | -0.01 (<0.01) |
| Concentration | Total motility after two days of storage | 0.01 (0.01) | -0.06 (0.07) | 0.33 (0.06) | -0.51 (0.12) | -0.02 (0.01) |
| Concentration | Progressive motility after two days of storage | 0.02 (0.01) | -0.04 (0.07) | 0.27 (0.06) | -0.63 (0.13) | 0.01 (<0.01) |
| Concentration | Total motility after three days of storage | 0.04 (0.01) | -0.13 (0.06) | 0.33 (0.05) | -0.27 (0.14) | 0.03 (<0.01) |
| Concentration | Progressive motility after three days of storage | 0.03 (0.01) | -0.12 (0.06) | 0.27 (0.05) | -0.28 (0.15) | 0.03 (<0.01) |
| Total number of sperm cells | Total number of normal sperm cells | 0.91 (<0.01) | 0.93 (0.01) | 0.91 (0.01) | 0.78 (0.09) | 0.91 (<0.01) |
| Total number of sperm cells | Total number of motile sperm cells | 0.98 (<0.01) | 0.98 (<0.01) | 0.98 (<0.01) | 0.94 (0.02) | 0.98 (<0.01) |
| Total number of sperm cells | Coiled Tail | -0.56 (0.01) | -0.77 (0.04) | -0.76 (0.03) | -0.84 (0.10) | -0.44 (<0.01) |
| Total number of sperm cells | Bent Tail | -0.18 (0.01) | -0.08 (0.09) | -0.31 (0.05) | -0.20 (0.31) | -0.18 (<0.01) |
| Total number of sperm cells | Distal Midpiece Reflex | 0.07 (0.01) | 0.29 (0.09) | 0.01 (0.05) | -0.34 (0.33) | 0.01 (<0.01) |
| Total number of sperm cells | Proximal cytoplasmatic droplets | -0.09 (0.01) | -0.20 (0.09) | -0.22 (0.05) | 0.59 (0.19) | -0.02 (<0.01) |
| Total number of sperm cells | Distal cytoplasmatic droplets | 0.07 (0.01) | 0.05 (0.09) | 0.01 (0.06) | 0.33 (0.23) | 0.10 (<0.01) |
| Total number of sperm cells | Total cytoplasmatic droplets | -0.04 (0.01) | -0.10 (0.09) | -0.16 (0.05) | 0.50 (0.20) | 0.03 (<0.01) |
| Total number of sperm cells | Abnormal Head | -0.07 (0.01) | -0.09 (0.06) | -0.19 (0.04) | -0.08 (0.22) | -0.04 (<0.01) |
| Total number of sperm cells | Abnormal Acrosome | -0.09 (0.02) | -0.14 (0.12) | -0.27 (0.14) | -0.23 (0.23) | -0.04 (0.02) |
| Total number of sperm cells | Total morphological abnormalities | 0.01 (0.01) | 0.01 (0.07) | -0.08 (0.04) | 0.28 (0.19) | 0.03 (<0.01) |
| Total number of sperm cells | Total motility of fresh semen | 0.16 (0.01) | 0.06 (0.07) | 0.28 (0.03) | -0.10 (0.19) | 0.15 (<0.01) |
| Total number of sperm cells | Progressive motility of fresh semen | 0.14 (0.01) | 0.09 (0.06) | 0.28 (0.04) | 0.10 (0.18) | 0.11 (<0.01) |
| Total number of sperm cells | Total motility after one day of storage | 0.02 (0.01) | -0.13 (0.07) | 0.29 (0.06) | 0.56 (0.25) | -0.02 (<0.01) |
| Total number of sperm cells | Progressive motility after one day of storage | 0.01 (0.01) | -0.09 (0.07) | 0.20 (0.06) | 0.32 (0.23) | -0.02 (<0.01) |
| Total number of sperm cells | Total motility after two days of storage | 0.03 (0.01) | -0.01 (0.07) | 0.27 (0.06) | 0.07 (0.20) | -0.02 (<0.01) |
| Total number of sperm cells | Progressive motility after two days of storage | 0.05 (0.01) | 0.05 (0.07) | 0.20 (0.06) | 0.30 (0.22) | 0.01 (<0.01) |
| Total number of sperm cells | Total motility after three days of storage | 0.04 (0.01) | -0.07 (0.06) | 0.33 (0.04) | 0.03 (0.20) | -0.01 (<0.01) |
| Total number of sperm cells | Progressive motility after three days of storage | 0.04 (0.01) | -0.09 (0.06) | 0.29 (0.04) | 0.13 (0.21) | 0.01 (<0.01) |
| Total number of normal sperm cells | Total number of motile sperm cells | 0.94 (<0.01) | 0.96 (0.01) | 0.95 (<0.01) | 0.85 (0.06) | 0.94 (<0.01) |
| Total number of normal sperm cells | Coiled Tail | -0.50 (0.01) | -0.73 (0.05) | -0.71 (0.03) | -0.58 (0.21) | -0.39 (0.01) |
| Total number of normal sperm cells | Bent Tail | -0.30 (0.01) | -0.34 (0.08) | -0.48 (0.04) | -0.99 (0.15) | -0.24 (<0.01) |
| Total number of normal sperm cells | Distal Midpiece Reflex | -0.15 (0.01) | -0.05 (0.09) | -0.24 (0.05) | -1.00 (0.19) | -0.13 (<0.01) |
| Total number of normal sperm cells | Proximal cytoplasmatic droplets | -0.32 (0.01) | -0.49 (0.07) | -0.48 (0.04) | -0.49 (0.22) | -0.17 (<0.01) |
| Total number of normal sperm cells | Distal cytoplasmatic droplets | -0.16 (0.01) | -0.28 (0.09) | -0.27 (0.05) | -0.66 (0.21) | -0.05 (<0.01) |
| Total number of normal sperm cells | Total cytoplasmatic droplets | -0.30 (0.01) | -0.46 (0.08) | -0.45 (0.04) | -0.62 (0.19) | -0.17 (<0.01) |
| Total number of normal sperm cells | Abnormal Head | -0.14 (0.01) | -0.18 (0.06) | -0.31 (0.04) | -0.34 (0.23) | -0.09 (<0.01) |
| Total number of normal sperm cells | Abnormal Acrosome | -0.11 (0.02) | -0.20 (0.12) | -0.31 (0.14) | -0.34 (0.26) | -0.05 (0.02) |
| Total number of normal sperm cells | Total morphological abnormalities | -0.33 (0.01) | -0.37 (0.06) | -0.47 (0.03) | -0.39 (0.19) | -0.26 (<0.01) |
| Total number of normal sperm cells | Total motility of fresh semen | 0.30 (0.01) | 0.27 (0.06) | 0.50 (0.03) | 0.40 (0.21) | 0.22 (<0.01) |
| Total number of normal sperm cells | Progressive motility of fresh semen | 0.31 (0.01) | 0.30 (0.06) | 0.51 (0.03) | 0.78 (0.15) | 0.23 (<0.01) |
| Total number of normal sperm cells | Total motility after one day of storage | 0.13 (0.01) | -0.04 (0.08) | 0.50 (0.06) | 0.66 (0.24) | 0.07 (0.01) |
| Total number of normal sperm cells | Progressive motility after one day of storage | 0.13 (0.01) | 0.04 (0.08) | 0.44 (0.06) | 0.62 (0.22) | 0.07 (0.01) |
| Total number of normal sperm cells | Total motility after two days of storage | 0.15 (0.01) | 0.09 (0.07) | 0.50 (0.05) | 0.43 (0.21) | 0.07 (0.01) |
| Total number of normal sperm cells | Progressive motility after two days of storage | 0.18 (0.01) | 0.15 (0.07) | 0.45 (0.05) | 0.50 (0.23) | 0.10 (0.01) |
| Total number of normal sperm cells | Total motility after three days of storage | 0.21 (0.01) | 0.05 (0.06) | 0.59 (0.04) | 0.14 (0.22) | 0.15 (<0.01) |
| Total number of normal sperm cells | Progressive motility after three days of storage | 0.23 (0.01) | 0.06 (0.06) | 0.56 (0.04) | 0.36 (0.21) | 0.17 (<0.01) |
| Total number of motile sperm cells | Coiled Tail | -0.56 (0.01) | -0.79 (0.04) | -0.76 (0.02) | -0.84 (0.09) | -0.44 (<0.01) |
| Total number of motile sperm cells | Bent Tail | -0.29 (0.01) | -0.26 (0.09) | -0.43 (0.04) | -0.52 (0.26) | -0.26 (<0.01) |
| Total number of motile sperm cells | Distal Midpiece Reflex | -0.05 (0.01) | 0.10 (0.09) | -0.13 (0.05) | -0.44 (0.29) | -0.07 (<0.01) |
| Total number of motile sperm cells | Proximal cytoplasmatic droplets | -0.18 (0.01) | -0.32 (0.08) | -0.33 (0.05) | 0.29 (0.22) | -0.08 (<0.01) |
| Total number of motile sperm cells | Distal cytoplasmatic droplets | -0.04 (0.01) | -0.11 (0.09) | -0.13 (0.05) | 0.11 (0.24) | 0.02 (<0.01) |
| Total number of motile sperm cells | Total cytoplasmatic droplets | -0.15 (0.01) | -0.26 (0.09) | -0.28 (0.05) | 0.21 (0.23) | -0.06 (<0.01) |
| Total number of motile sperm cells | Abnormal Head | -0.09 (0.01) | -0.13 (0.06) | -0.23 (0.04) | -0.23 (0.20) | -0.05 (<0.01) |
| Total number of motile sperm cells | Abnormal Acrosome | -0.08 (0.02) | -0.13 (0.12) | -0.29 (0.14) | -0.34 (0.22) | -0.04 (0.02) |
| Total number of motile sperm cells | Total morphological abnormalities | -0.10 (0.01) | -0.13 (0.07) | -0.21 (0.03) | 0.10 (0.19) | -0.05 (<0.01) |
| Total number of motile sperm cells | Total motility of fresh semen | 0.32 (0.01) | 0.25 (0.06) | 0.45 (0.03) | 0.23 (0.18) | 0.29 (<0.01) |
| Total number of motile sperm cells | Progressive motility of fresh semen | 0.29 (0.01) | 0.26 (0.06) | 0.43 (0.03) | 0.37 (0.16) | 0.24 (<0.01) |
| Total number of motile sperm cells | Total motility after one day of storage | 0.08 (0.01) | -0.02 (0.07) | 0.38 (0.06) | 0.61 (0.23) | 0.02 (0.01) |
| Total number of motile sperm cells | Progressive motility after one day of storage | 0.07 (0.01) | 0.02 (0.07) | 0.29 (0.06) | 0.36 (0.22) | 0.02 (0.01) |
| Total number of motile sperm cells | Total motility after two days of storage | 0.11 (0.01) | 0.08 (0.07) | 0.41 (0.06) | 0.13 (0.20) | 0.03 (<0.01) |
| Total number of motile sperm cells | Progressive motility after two days of storage | 0.12 (0.01) | 0.14 (0.07) | 0.34 (0.05) | 0.33 (0.22) | 0.05 (<0.01) |
| Total number of motile sperm cells | Total motility after three days of storage | 0.12 (0.01) | 0.05 (0.06) | 0.45 (0.04) | -0.03 (0.20) | 0.05 (<0.01) |
| Total number of motile sperm cells | Progressive motility after three days of storage | 0.13 (0.01) | 0.04 (0.06) | 0.42 (0.04) | 0.15 (0.20) | 0.06 (<0.01) |
| Coiled Tail | Bent Tail | 0.20 (0.01) | 0.23 (0.10) | 0.43 (0.05) | 0.66 (0.17) | 0.14 (0.01) |
| Coiled Tail | Distal Midpiece Reflex | -0.03 (0.01) | -0.15 (0.10) | -0.04 (0.06) | 0.17 (0.29) | 0.01 (0.01) |
| Coiled Tail | Proximal cytoplasmatic droplets | 0.09 (0.01) | 0.30 (0.10) | 0.16 (0.06) | 0.02 (0.23) | <0.01 (0.01) |
| Coiled Tail | Distal cytoplasmatic droplets | -0.04 (0.01) | -0.01 (0.10) | -0.01 (0.06) | 0.05 (0.23) | -0.07 (0.01) |
| Coiled Tail | Total cytoplasmatic droplets | 0.04 (0.01) | 0.19 (0.11) | 0.10 (0.06) | 0.06 (0.22) | -0.03 (0.01) |
| Coiled Tail | Abnormal Head | 0.05 (0.01) | 0.15 (0.10) | 0.10 (0.06) | -0.06 (0.24) | 0.02 (0.01) |
| Coiled Tail | Abnormal Acrosome | 0.06 (0.04) | 0.29 (0.19) | 0.12 (0.19) | 0.53 (0.25) | -0.01 (0.05) |
| Coiled Tail | Total morphological abnormalities | -0.02 (0.01) | -0.04 (0.10) | 0.03 (0.06) | -0.54 (0.19) | -0.02 (0.01) |
| Coiled Tail | Total motility of fresh semen | -0.18 (0.01) | -0.14 (0.10) | -0.31 (0.05) | -0.50 (0.19) | -0.14 (0.01) |
| Coiled Tail | Progressive motility of fresh semen | -0.15 (0.01) | -0.15 (0.09) | -0.30 (0.05) | -0.39 (0.19) | -0.10 (0.01) |
| Coiled Tail | Total motility after one day of storage | -0.01 (0.02) | 0.05 (0.11) | -0.26 (0.10) | -0.24 (0.33) | 0.03 (0.02) |
| Coiled Tail | Progressive motility after one day of storage | -0.03 (0.02) | -0.03 (0.11) | -0.19 (0.09) | 0.05 (0.28) | 0.01 (0.02) |
| Coiled Tail | Total motility after two days of storage | -0.04 (0.01) | -0.13 (0.10) | -0.18 (0.08) | 0.02 (0.22) | 0.01 (0.01) |
| Coiled Tail | Progressive motility after two days of storage | -0.07 (0.01) | -0.23 (0.10) | -0.15 (0.08) | -0.12 (0.24) | -0.01 (0.01) |
| Coiled Tail | Total motility after three days of storage | -0.04 (0.01) | 0.05 (0.10) | -0.35 (0.07) | 0.74 (0.19) | <0.01 (0.01) |
| Coiled Tail | Progressive motility after three days of storage | -0.06 (0.01) | -0.02 (0.10) | -0.32 (0.07) | 0.45 (0.24) | -0.02 (0.01) |
| Bent Tail | Distal Midpiece Reflex | 0.40 (0.01) | 0.74 (0.05) | 0.60 (0.03) | 0.56 (0.20) | 0.22 (<0.01) |
| Bent Tail | Proximal cytoplasmatic droplets | 0.37 (0.01) | 0.49 (0.08) | 0.59 (0.04) | 0.92 (0.04) | 0.24 (<0.01) |
| Bent Tail | Distal cytoplasmatic droplets | 0.33 (0.01) | 0.41 (0.08) | 0.53 (0.04) | 0.80 (0.09) | 0.22 (<0.01) |
| Bent Tail | Total cytoplasmatic droplets | 0.40 (0.01) | 0.51 (0.08) | 0.63 (0.03) | 0.89 (0.05) | 0.28 (<0.01) |
| Bent Tail | Abnormal Head | 0.08 (0.01) | 0.30 (0.09) | 0.14 (0.05) | 0.25 (0.25) | 0.03 (<0.01) |
| Bent Tail | Abnormal Acrosome | 0.04 (0.02) | 0.36 (0.17) | 0.19 (0.19) | 0.68 (0.20) | -0.05 (0.02) |
| Bent Tail | Total morphological abnormalities | 0.39 (0.01) | 0.67 (0.06) | 0.58 (0.03) | 0.60 (0.15) | 0.26 (<0.01) |
| Bent Tail | Total motility of fresh semen | -0.55 (0.01) | -0.79 (0.04) | -0.79 (0.02) | -0.99 (0.04) | -0.40 (<0.01) |
| Bent Tail | Progressive motility of fresh semen | -0.51 (0.01) | -0.79 (0.04) | -0.78 (0.02) | -0.99 (0.04) | -0.36 (<0.01) |
| Bent Tail | Total motility after one day of storage | -0.20 (0.01) | -0.24 (0.10) | -0.70 (0.06) | 0.20 (0.33) | -0.07 (0.01) |
| Bent Tail | Progressive motility after one day of storage | -0.22 (0.01) | -0.44 (0.09) | -0.61 (0.06) | 0.18 (0.29) | -0.09 (0.01) |
| Bent Tail | Total motility after two days of storage | -0.24 (0.01) | -0.34 (0.08) | -0.70 (0.05) | -0.59 (0.16) | -0.10 (0.01) |
| Bent Tail | Progressive motility after two days of storage | -0.25 (0.01) | -0.45 (0.08) | -0.67 (0.05) | -0.57 (0.18) | -0.10 (0.01) |
| Bent Tail | Total motility after three days of storage | -0.27 (0.01) | -0.45 (0.07) | -0.68 (0.04) | 0.49 (0.16) | -0.15 (<0.01) |
| Bent Tail | Progressive motility after three days of storage | -0.32 (0.01) | -0.54 (0.06) | -0.69 (0.04) | -0.01 (0.23) | -0.18 (<0.01) |
| Distal Midpiece Reflex | Proximal cytoplasmatic droplets | 0.33 (0.01) | 0.34 (0.09) | 0.37 (0.04) | 0.85 (0.10) | 0.24 (<0.01) |
| Distal Midpiece Reflex | Distal cytoplasmatic droplets | 0.46 (0.01) | 0.57 (0.07) | 0.53 (0.04) | 0.76 (0.13) | 0.32 (<0.01) |
| Distal Midpiece Reflex | Total cytoplasmatic droplets | 0.44 (0.01) | 0.48 (0.08) | 0.49 (0.04) | 0.83 (0.10) | 0.34 (<0.01) |
| Distal Midpiece Reflex | Abnormal Head | 0.01 (0.01) | 0.14 (0.09) | -0.10 (0.05) | 0.24 (0.26) | 0.01 (<0.01) |
| Distal Midpiece Reflex | Abnormal Acrosome | 0.01 (0.03) | 0.27 (0.17) | -0.39 (0.20) | 0.65 (0.23) | 0.01 (0.02) |
| Distal Midpiece Reflex | Total morphological abnormalities | 0.61 (0.01) | 0.63 (0.05) | 0.73 (0.02) | 0.82 (0.11) | 0.49 (<0.01) |
| Distal Midpiece Reflex | Total motility of fresh semen | -0.56 (0.01) | -0.66 (0.05) | -0.66 (0.03) | -0.66 (0.14) | -0.40 (<0.01) |
| Distal Midpiece Reflex | Progressive motility of fresh semen | -0.56 (0.01) | -0.70 (0.04) | -0.68 (0.03) | -0.61 (0.15) | -0.41 (<0.01) |
| Distal Midpiece Reflex | Total motility after one day of storage | -0.34 (0.01) | -0.35 (0.08) | -0.70 (0.05) | -0.29 (0.33) | -0.19 (0.01) |
| Distal Midpiece Reflex | Progressive motility after one day of storage | -0.37 (0.01) | -0.46 (0.08) | -0.69 (0.05) | -0.36 (0.28) | -0.20 (0.01) |
| Distal Midpiece Reflex | Total motility after two days of storage | -0.36 (0.01) | -0.35 (0.08) | -0.71 (0.05) | -0.77 (0.14) | -0.19 (0.01) |
| Distal Midpiece Reflex | Progressive motility after two days of storage | -0.38 (0.01) | -0.44 (0.07) | -0.70 (0.04) | -0.88 (0.11) | -0.21 (0.01) |
| Distal Midpiece Reflex | Total motility after three days of storage | -0.36 (0.01) | -0.43 (0.07) | -0.62 (0.04) | 0.05 (0.23) | -0.22 (<0.01) |
| Distal Midpiece Reflex | Progressive motility after three days of storage | -0.42 (0.01) | -0.56 (0.06) | -0.61 (0.04) | -0.48 (0.22) | -0.25 (<0.01) |
| Proximal cytoplasmatic droplets | Distal cytoplasmatic droplets | 0.51 (0.01) | 0.48 (0.07) | 0.64 (0.04) | 0.98 (0.02) | 0.37 (<0.01) |
| Proximal cytoplasmatic droplets | Total cytoplasmatic droplets | 0.89 (<0.01) | 0.88 (0.02) | 0.93 (0.01) | 1.00 (<0.01) | 0.83 (<0.01) |
| Proximal cytoplasmatic droplets | Abnormal Head | 0.14 (0.01) | 0.30 (0.08) | 0.24 (0.05) | 0.53 (0.15) | 0.06 (<0.01) |
| Proximal cytoplasmatic droplets | Abnormal Acrosome | 0.13 (0.03) | 0.50 (0.17) | 0.03 (0.18) | 0.37 (0.22) | 0.03 (0.02) |
| Proximal cytoplasmatic droplets | Total morphological abnormalities | 0.69 (0.01) | 0.80 (0.03) | 0.79 (0.02) | 0.87 (0.05) | 0.55 (<0.01) |
| Proximal cytoplasmatic droplets | Total motility of fresh semen | -0.46 (0.01) | -0.51 (0.07) | -0.61 (0.03) | -0.75 (0.09) | -0.28 (<0.01) |
| Proximal cytoplasmatic droplets | Progressive motility of fresh semen | -0.44 (0.01) | -0.48 (0.06) | -0.64 (0.03) | -0.85 (0.06) | -0.24 (<0.01) |
| Proximal cytoplasmatic droplets | Total motility after one day of storage | -0.12 (0.02) | -0.10 (0.10) | -0.32 (0.08) | -0.12 (0.26) | -0.06 (0.01) |
| Proximal cytoplasmatic droplets | Progressive motility after one day of storage | -0.18 (0.02) | -0.30 (0.10) | -0.32 (0.07) | -0.14 (0.21) | -0.08 (0.01) |
| Proximal cytoplasmatic droplets | Total motility after two days of storage | -0.23 (0.02) | -0.16 (0.09) | -0.51 (0.06) | -0.67 (0.11) | -0.09 (0.01) |
| Proximal cytoplasmatic droplets | Progressive motility after two days of storage | -0.29 (0.01) | -0.36 (0.08) | -0.53 (0.05) | -0.66 (0.12) | -0.12 (0.01) |
| Proximal cytoplasmatic droplets | Total motility after three days of storage | -0.25 (0.01) | -0.32 (0.08) | -0.53 (0.05) | 0.07 (0.17) | -0.13 (<0.01) |
| Proximal cytoplasmatic droplets | Progressive motility after three days of storage | -0.33 (0.01) | -0.41 (0.07) | -0.57 (0.04) | -0.54 (0.14) | -0.17 (<0.01) |
| Distal cytoplasmatic droplets | Total cytoplasmatic droplets | 0.83 (<0.01) | 0.83 (0.03) | 0.87 (0.01) | 0.99 (0.01) | 0.80 (<0.01) |
| Distal cytoplasmatic droplets | Abnormal Head | 0.09 (0.01) | 0.27 (0.08) | 0.12 (0.06) | 0.39 (0.19) | 0.03 (<0.01) |
| Distal cytoplasmatic droplets | Abnormal Acrosome | 0.04 (0.03) | 0.44 (0.16) | -0.37 (0.22) | 0.10 (0.26) | 0.01 (0.02) |
| Distal cytoplasmatic droplets | Total morphological abnormalities | 0.68 (0.01) | 0.81 (0.03) | 0.75 (0.02) | 0.81 (0.08) | 0.55 (<0.01) |
| Distal cytoplasmatic droplets | Total motility of fresh semen | -0.50 (0.01) | -0.63 (0.06) | -0.62 (0.03) | -0.58 (0.13) | -0.34 (<0.01) |
| Distal cytoplasmatic droplets | Progressive motility of fresh semen | -0.51 (0.01) | -0.56 (0.05) | -0.69 (0.03) | -0.68 (0.10) | -0.36 (<0.01) |
| Distal cytoplasmatic droplets | Total motility after one day of storage | -0.30 (0.01) | -0.49 (0.08) | -0.40 (0.07) | 0.05 (0.26) | -0.20 (0.01) |
| Distal cytoplasmatic droplets | Progressive motility after one day of storage | -0.31 (0.01) | -0.55 (0.07) | -0.41 (0.07) | -0.09 (0.22) | -0.20 (0.01) |
| Distal cytoplasmatic droplets | Total motility after two days of storage | -0.36 (0.01) | -0.50 (0.07) | -0.50 (0.06) | -0.71 (0.10) | -0.22 (0.01) |
| Distal cytoplasmatic droplets | Progressive motility after two days of storage | -0.38 (0.01) | -0.58 (0.06) | -0.52 (0.05) | -0.78 (0.10) | -0.22 (0.01) |
| Distal cytoplasmatic droplets | Total motility after three days of storage | -0.35 (0.01) | -0.59 (0.06) | -0.48 (0.05) | 0.27 (0.17) | -0.23 (<0.01) |
| Distal cytoplasmatic droplets | Progressive motility after three days of storage | -0.41 (0.01) | -0.62 (0.05) | -0.55 (0.04) | -0.28 (0.19) | -0.26 (<0.01) |
| Total cytoplasmatic droplets | Abnormal Head | 0.14 (0.01) | 0.34 (0.09) | 0.20 (0.05) | 0.50 (0.16) | 0.05 (<0.01) |
| Total cytoplasmatic droplets | Abnormal Acrosome | 0.11 (0.03) | 0.59 (0.16) | -0.15 (0.19) | 0.31 (0.23) | 0.01 (0.02) |
| Total cytoplasmatic droplets | Total morphological abnormalities | 0.80 (<0.01) | 0.92 (0.02) | 0.86 (0.01) | 0.87 (0.04) | 0.69 (<0.01) |
| Total cytoplasmatic droplets | Total motility of fresh semen | -0.55 (0.01) | -0.65 (0.06) | -0.66 (0.03) | -0.70 (0.09) | -0.37 (<0.01) |
| Total cytoplasmatic droplets | Progressive motility of fresh semen | -0.54 (0.01) | -0.59 (0.05) | -0.73 (0.03) | -0.80 (0.07) | -0.36 (<0.01) |
| Total cytoplasmatic droplets | Total motility after one day of storage | -0.25 (0.02) | -0.34 (0.09) | -0.42 (0.07) | -0.09 (0.25) | -0.17 (0.01) |
| Total cytoplasmatic droplets | Progressive motility after one day of storage | -0.30 (0.02) | -0.48 (0.08) | -0.44 (0.07) | -0.17 (0.21) | -0.19 (0.01) |
| Total cytoplasmatic droplets | Total motility after two days of storage | -0.35 (0.01) | -0.40 (0.08) | -0.58 (0.05) | -0.71 (0.10) | -0.20 (0.01) |
| Total cytoplasmatic droplets | Progressive motility after two days of storage | -0.40 (0.01) | -0.55 (0.07) | -0.60 (0.05) | -0.77 (0.09) | -0.23 (0.01) |
| Total cytoplasmatic droplets | Total motility after three days of storage | -0.34 (0.01) | -0.54 (0.06) | -0.56 (0.04) | 0.18 (0.17) | -0.22 (<0.01) |
| Total cytoplasmatic droplets | Progressive motility after three days of storage | -0.43 (0.01) | -0.60 (0.06) | -0.62 (0.04) | -0.46 (0.15) | -0.27 (<0.01) |
| Abnormal Head | Abnormal Acrosome | 0.11 (0.02) | 0.31 (0.11) | 0.31 (0.16) | 0.23 (0.21) | 0.05 (0.02) |
| Abnormal Head | Total morphological abnormalities | 0.23 (0.01) | 0.30 (0.06) | 0.36 (0.04) | 0.49 (0.14) | 0.18 (<0.01) |
| Abnormal Head | Total motility of fresh semen | -0.15 (0.01) | -0.29 (0.06) | -0.33 (0.04) | -0.56 (0.13) | -0.05 (<0.01) |
| Abnormal Head | Progressive motility of fresh semen | -0.14 (0.01) | -0.25 (0.06) | -0.30 (0.04) | -0.57 (0.12) | -0.06 (<0.01) |
| Abnormal Head | Total motility after one day of storage | -0.07 (0.01) | -0.06 (0.08) | -0.34 (0.07) | -0.49 (0.20) | -0.02 (0.01) |
| Abnormal Head | Progressive motility after one day of storage | -0.08 (0.01) | -0.11 (0.07) | -0.29 (0.06) | -0.50 (0.16) | -0.02 (0.01) |
| Abnormal Head | Total motility after two days of storage | -0.05 (0.01) | -0.05 (0.07) | -0.26 (0.07) | -0.20 (0.17) | -0.01 (0.01) |
| Abnormal Head | Progressive motility after two days of storage | -0.06 (0.01) | -0.06 (0.07) | -0.27 (0.06) | -0.41 (0.18) | -0.02 (0.01) |
| Abnormal Head | Total motility after three days of storage | -0.10 (0.01) | -0.14 (0.06) | -0.34 (0.05) | -0.41 (0.15) | -0.04 (<0.01) |
| Abnormal Head | Progressive motility after three days of storage | -0.12 (0.01) | -0.16 (0.06) | -0.34 (0.05) | -0.55 (0.14) | -0.05 (<0.01) |
| Abnormal Acrosome | Total morphological abnormalities | 0.13 (0.02) | 0.25 (0.12) | 0.15 (0.14) | 0.28 (0.18) | 0.10 (0.02) |
| Abnormal Acrosome | Total motility of fresh semen | -0.03 (0.02) | 0.08 (0.13) | -0.23 (0.16) | -0.40 (0.17) | 0.01 (0.02) |
| Abnormal Acrosome | Progressive motility of fresh semen | -0.05 (0.02) | -0.14 (0.12) | -0.08 (0.15) | -0.44 (0.16) | 0.01 (0.02) |
| Abnormal Acrosome | Total motility after one day of storage | 0.06 (0.04) | 0.29 (0.14) | -0.39 (0.21) | -0.17 (0.29) | 0.08 (0.05) |
| Abnormal Acrosome | Progressive motility after one day of storage | 0.07 (0.04) | 0.17 (0.14) | -0.23 (0.22) | -0.07 (0.24) | 0.09 (0.05) |
| Abnormal Acrosome | Total motility after two days of storage | 0.04 (0.03) | 0.26 (0.13) | -0.28 (0.21) | -0.05 (0.19) | 0.04 (0.04) |
| Abnormal Acrosome | Progressive motility after two days of storage | 0.03 (0.03) | 0.23 (0.13) | -0.13 (0.21) | -0.26 (0.21) | 0.02 (0.04) |
| Abnormal Acrosome | Total motility after three days of storage | -0.02 (0.02) | 0.36 (0.12) | -0.61 (0.19) | 0.06 (0.18) | -0.03 (0.02) |
| Abnormal Acrosome | Progressive motility after three days of storage | -0.03 (0.02) | 0.24 (0.12) | -0.53 (0.20) | 0.01 (0.20) | -0.03 (0.02) |
| Total morphological abnormalities | Total motility of fresh semen | -0.49 (0.01) | -0.62 (0.04) | -0.68 (0.02) | -0.61 (0.09) | -0.27 (<0.01) |
| Total morphological abnormalities | Progressive motility of fresh semen | -0.53 (0.01) | -0.62 (0.04) | -0.75 (0.02) | -0.78 (0.06) | -0.33 (<0.01) |
| Total morphological abnormalities | Total motility after one day of storage | -0.40 (0.01) | -0.41 (0.06) | -0.70 (0.04) | -0.23 (0.19) | -0.29 (0.01) |
| Total morphological abnormalities | Progressive motility after one day of storage | -0.45 (0.01) | -0.53 (0.05) | -0.72 (0.04) | -0.36 (0.15) | -0.32 (0.01) |
| Total morphological abnormalities | Total motility after two days of storage | -0.37 (0.01) | -0.37 (0.06) | -0.75 (0.04) | -0.64 (0.12) | -0.21 (0.01) |
| Total morphological abnormalities | Progressive motility after two days of storage | -0.42 (0.01) | -0.43 (0.06) | -0.77 (0.03) | -0.60 (0.14) | -0.27 (0.01) |
| Total morphological abnormalities | Total motility after three days of storage | -0.39 (0.01) | -0.44 (0.05) | -0.73 (0.02) | -0.33 (0.13) | -0.22 (<0.01) |
| Total morphological abnormalities | Progressive motility after three days of storage | -0.44 (0.01) | -0.52 (0.05) | -0.74 (0.02) | -0.63 (0.10) | -0.27 (<0.01) |
| Total motility of fresh semen | Progressive motility of fresh semen | 0.83 (<0.01) | 0.83 (0.02) | 0.94 (0.01) | 0.81 (0.05) | 0.75 (<0.01) |
| Total motility of fresh semen | Total motility after one day of storage | 0.46 (0.01) | 0.72 (0.04) | 0.82 (0.02) | 0.40 (0.17) | 0.16 (<0.01) |
| Total motility of fresh semen | Progressive motility after one day of storage | 0.46 (0.01) | 0.76 (0.04) | 0.77 (0.02) | 0.29 (0.16) | 0.16 (<0.01) |
| Total motility of fresh semen | Total motility after two days of storage | 0.47 (0.01) | 0.71 (0.04) | 0.85 (0.03) | 0.35 (0.14) | 0.17 (<0.01) |
| Total motility of fresh semen | Progressive motility after two days of storage | 0.46 (0.01) | 0.70 (0.04) | 0.82 (0.02) | 0.37 (0.16) | 0.18 (<0.01) |
| Total motility of fresh semen | Total motility after three days of storage | 0.41 (0.01) | 0.70 (0.03) | 0.74 (0.02) | -0.23 (0.15) | 0.15 (<0.01) |
| Total motility of fresh semen | Progressive motility after three days of storage | 0.44 (0.01) | 0.71 (0.03) | 0.75 (0.02) | 0.04 (0.16) | 0.18 (<0.01) |
| Progressive motility of fresh semen | Total motility after one day of storage | 0.42 (0.01) | 0.52 (0.05) | 0.80 (0.03) | 0.57 (0.15) | 0.22 (<0.01) |
| Progressive motility of fresh semen | Progressive motility after one day of storage | 0.50 (0.01) | 0.75 (0.03) | 0.85 (0.02) | 0.60 (0.12) | 0.25 (<0.01) |
| Progressive motility of fresh semen | Total motility after two days of storage | 0.43 (0.01) | 0.55 (0.05) | 0.84 (0.03) | 0.36 (0.13) | 0.23 (<0.01) |
| Progressive motility of fresh semen | Progressive motility after two days of storage | 0.51 (0.01) | 0.76 (0.03) | 0.90 (0.02) | 0.35 (0.14) | 0.26 (<0.01) |
| Progressive motility of fresh semen | Total motility after three days of storage | 0.41 (0.01) | 0.55 (0.04) | 0.77 (0.02) | -0.12 (0.14) | 0.23 (<0.01) |
| Progressive motility of fresh semen | Progressive motility after three days of storage | 0.50 (0.01) | 0.69 (0.03) | 0.82 (0.02) | 0.34 (0.13) | 0.29 (<0.01) |
| Total motility after one day of storage | Progressive motility after one day of storage | 0.84 (<0.01) | 0.90 (0.02) | 0.92 (0.02) | 0.95 (0.03) | 0.80 (<0.01) |
| Total motility after one day of storage | Total motility after two days of storage | 0.62 (0.01) | 0.97 (0.01) | 0.96 (0.02) | 0.74 (0.10) | 0.43 (0.01) |
| Total motility after one day of storage | Progressive motility after two days of storage | 0.53 (0.01) | 0.84 (0.03) | 0.86 (0.03) | 0.91 (0.07) | 0.35 (0.01) |
| Total motility after one day of storage | Total motility after three days of storage | 0.75 (<0.01) | 0.99 (0.01) | 0.91 (0.01) | 0.96 (0.03) | 0.60 (0.01) |
| Total motility after one day of storage | Progressive motility after three days of storage | 0.70 (<0.01) | 0.93 (0.01) | 0.88 (0.02) | 0.99 (0.04) | 0.56 (0.01) |
| Progressive motility after one day of storage | Total motility after two days of storage | 0.50 (0.01) | 0.79 (0.04) | 0.83 (0.04) | 0.69 (0.11) | 0.32 (0.01) |
| Progressive motility after one day of storage | Progressive motility after two days of storage | 0.57 (0.01) | 0.97 (0.01) | 0.98 (0.01) | 0.91 (0.05) | 0.34 (0.01) |
| Progressive motility after one day of storage | Total motility after three days of storage | 0.72 (<0.01) | 0.94 (0.01) | 0.89 (0.02) | 0.80 (0.07) | 0.57 (0.01) |
| Progressive motility after one day of storage | Progressive motility after three days of storage | 0.74 (<0.01) | 0.99 (0.01) | 0.93 (0.01) | 0.95 (0.03) | 0.58 (0.01) |
| Total motility after two days of storage | Progressive motility after two days of storage | 0.80 (<0.01) | 0.83 (0.02) | 0.91 (0.02) | 0.97 (0.02) | 0.76 (<0.01) |
| Total motility after two days of storage | Total motility after three days of storage | 0.71 (<0.01) | 0.98 (0.01) | 0.97 (0.01) | 0.55 (0.11) | 0.52 (0.01) |
| Total motility after two days of storage | Progressive motility after three days of storage | 0.61 (0.01) | 0.84 (0.02) | 0.92 (0.02) | 0.57 (0.12) | 0.43 (0.01) |
| Progressive motility after two days of storage | Total motility after three days of storage | 0.61 (0.01) | 0.87 (0.02) | 0.90 (0.02) | 0.81 (0.07) | 0.42 (0.01) |
| Progressive motility after two days of storage | Progressive motility after three days of storage | 0.66 (0.01) | 0.97 (0.01) | 0.98 (0.01) | 0.95 (0.03) | 0.44 (0.01) |
| Total motility after three days of storage | Progressive motility after three days of storage | 0.89 (<0.01) | 0.93 (0.01) | 0.96 (0.01) | 0.82 (0.05) | 0.85 (<0.01) |
