# Supplementary material for: Genetic parameters and parental and early-life effects of boar semen traits
Source: Genet Sel Evol. 2025 Feb 6;57:4. doi: 10.1186/s12711-025-00954-6 (PMC11800458; doi:10.1186/s12711-025-00954-6)
Supplement: Supplementary file 4 — Additional file 4: Table S4 Significance of early-life conditions on semen production traits. [file 12711_2025_954_MOESM4_ESM.docx]

**Significance of early-life conditions on semen production traits.**

| **Trait** | **Age of the dam** | **Age of the sire** | **Dam parity** | **Gestation length** | **Litter size** | **Litter sex ratio** | **Number of born alive** | **Mates at weaning** | **Rearing length** | **Rearing weight growth** |
| --- | --- | --- | --- | --- | --- | --- | --- | --- | --- | --- |
| **Semen Quantity** | | | | | | | | | | |
| Volume (mL) | - | 2.4 | - | - | - | - | - | - | - | - |
| Concentration (10^6^/mL) | - | 1.4 | - | - | - | - | - | - | 1.4 | - |
| Total number of sperm cells (10^9^) | - | - | - | - | - | - | - | - | - | - |
| Total number of normal sperm cells (10^9^) | - | - | - | - | - | - | - | - | - | - |
| Total number of motile sperm cells (10^9^) | - | - | - | - | - | - | - | - | - | - |
| **Sperm Motility ^a)^** | | | | | | | | | | |
| Total motility of fresh semen | - | - | - | - | - | - | - | - | 1.5 | - |
| Total motility after 1 day of storage | - | - | - | - | - | - | - | - | - | - |
| Total motility after 2 days of storage | - | - | - | - | - | - | - | - | 1.8 | - |
| Total motility after 3 days of storage | - | - | - | - | 1.5 | - | - | - | - | - |
| Progressive motility of fresh semen | - | - | - | - | - | - | - | - | - | - |
| Progressive motility after 1 day of storage | - | - | - | - | - | - | - | - | - | - |
| Progressive motility after 2 days of storage | - | - | - | 1.5 | - | - | - | - | 1.6 | - |
| Progressive motility after 3 days of storage | - | - | - | - | 1.6 | - | - | - | - | - |
| **Sperm Morphology ^a)^** | | | | | | | | | | |
| Total morphological abnormalities | - | - | - | - | 1.4 | - | - | - | - | - |
| Total cytoplasmatic droplets | 3.2 | - | - | - | - | - | - | - | - | 1.5 |
| Proximal cytoplasmatic droplets | 2.8 | - | - | - | - | - | - | - | - | - |
| Distal cytoplasmatic droplets | - | - | - | - | - | - | 2.7 | - | - | 1.5 |
| Distal Midpiece Reflex | - | - | 1.5 | - | - | - | 1.9 | - | 1.3 | - |
| Coiled Tail | - | - | - | - | - | - | - | - | - | - |
| Bent Tail | - | - | - | - | - | - | - | - | - | - |
| Abnormal Head | - | - | - | - | - | - | - | - | - | - |
| Abnormal Acrosome | - | - | - | 2.1 | - | - | - | - | - | - |

The p-values were log-transformed. Non-significant estimates are indicated with a dash. Significant early life effects are reported in bold. The remaining values are considered suggestive.

1. Early life effects were estimates for transformed trait values.
