# Supplementary material for: Genetic parameters and parental and early-life effects of boar semen traits
Source: Genet Sel Evol. 2025 Feb 6;57:4. doi: 10.1186/s12711-025-00954-6 (PMC11800458; doi:10.1186/s12711-025-00954-6)
Supplement: Supplementary file 5 — Additional file 5: Table S5 Percentage of the phenotypic variance that can be explained by Mitochondria and Y chromosome. [file 12711_2025_954_MOESM5_ESM.docx]

**Percentage of the phenotypic variance that can be explained by Mitochondrial DNA and Y chromosome.**

| **Trait** | **% Mitochondrial DNA**  **Variance ^a)^** | **% Y chromosome**  **Variance ^a)^** |
| --- | --- | --- |
| **Semen Quantity** | | |
| Volume (mL) | - | - |
| Concentration (10^6^/mL) | - | 3.6 _(2.1)_ |
| Total number of sperm cells (10^9^) | - | - |
| Total number of normal sperm cells (10^9^) | - | - |
| Total number of motile sperm cells (10^9^) | - | - |
| **Sperm Motility ^b)^** | | |
| Total motility of fresh semen | - | - |
| Total motility after 1 day of storage | - | - |
| Total motility after 2 days of storage | 0.9 _(0.6)_ | - |
| Total motility after 3 days of storage | 0.2 _(0.2)_ | - |
| Progressive motility of fresh semen | - | - |
| Progressive motility after 1 day of storage | - | - |
| Progressive motility after 2 days of storage | 1.0 _(0.6)_ | 0.02 _(0.1)_ |
| Progressive motility after 3 days of storage | 0.04 _(0.1)_ | - |
| **Sperm Morphology ^b)^** | | |
| Total morphological abnormalities | - | - |
| Total cytoplasmatic droplets | - | - |
| Proximal cytoplasmatic droplets | - | - |
| Distal cytoplasmatic droplets | - | 0.1 _(0.2)_ |
| Distal Midpiece Reflex | - | - |
| Coiled Tail | - | - |
| Bent Tail | - | - |
| Abnormal Head | - | - |
| Abnormal Acrosome | 0.6 _(0.6)_ | 0.5 _(0.7)_ |

Standard errors are shown in subscript.

1. The percentage of mitochondria or Y chromosome variances with “-” are estimated to be < 0.01.
2. Mitochondria and Y chromosome variances were estimates for transformed trait values.
